# Integrated phenomic and genomic analyses unveil modes of altered phenotypic plasticity during wheat improvement

**DOI:** 10.1186/s13059-025-03740-1

**Published:** 2025-08-28

**Authors:** Linqian Han, Xiaoming Wang, Ryan Benke, Laura E. Tibbs-Cortes, Peng Zhao, Karen A. Sanguinet, Zhiwu Zhang, Shengbao Xu, Jianming Yu, Xianran Li

**Affiliations:** 1 Department of Crop and Soil Sciences, Washington State University, Pullman, WA 99164 USA; 2 State Key Laboratory for Crop Stress Resistance and High-Efficiency Production, College of Agronomy, Northwest A&F University, Yangling, Shaanxi 712100 China; 3 USDA-ARS, Wheat Health, Genetics, and Quality Research Unit, Pullman, WA 99164 USA; 4 Department of Agronomy, Iowa State University, Ames, IA 50011 USA; 5 USDA-ARS, Corn Insects and Crop Genetics Research Unit, Ames, IA 50011 USA

**Keywords:** Phenotypic plasticity, Wheat improvement, Environmental index, Reaction-norm parameters, GWAS, Genomic prediction, *Rht-B1*, *Rht-D1*, Pleiotropic effect

## Abstract

**Background:**

Wheat has a critical role in global food security. During the improvement of wheat from landraces to cultivars, a suite of traits has been modified for higher yields. However, changing patterns of wheat in response to different environmental conditions, or phenotypic plasticity, during this improvement remain to be elucidated.

**Results:**

We measure 17 agronomic traits for 406 wheat accessions consisting of landraces and cultivars in 10 environments. Analyses reveal varied contributions from genotype and environment to phenotypic variation across the evaluated traits. Using environmental indices identified by Critical Environmental Regressor through Informed Search (CERIS), we model the phenotypic values across environments of each accession with two reaction-norm parameters (intercept and slope). Genome Wide Association Studies (GWAS) identify loci significantly associated with variation in the two parameters, including *Ppd-D1* and two Green Revolution genes (*Rht-D1* and *Rht-B1*). Compared with the corresponding wild-type allele, *Rht-D1b* alters intercept and slope of more traits than *Rht-B1b*. Among nine possible modes of phenotypic plasticity change from landraces to cultivars, three predominant modes account for 88% of evaluated traits. Generally, two reaction-norm parameters decrease simultaneously for plant architecture traits but increase simultaneously for yield component traits.

**Conclusions:**

We systematically evaluate phenome-wide wheat phenotypic plasticity. Two reaction-norm parameters based on specific environmental indices capture varied degrees of phenotypic plasticity for each trait across wheat accessions. Two Green Revolution genes have different effect spectra in altering phenome-wide phenotypic plasticity. By incorporating the evolutionary dimension, we reveal dominant modes of phenotypic plasticity change during wheat improvement.

**Supplementary Information:**

The online version contains supplementary material available at 10.1186/s13059-025-03740-1.

## Background

Wheat is an essential crop for global food security, contributing 55% of the carbohydrates, 21% of the total protein, and 19–20% of the calories consumed in human diets worldwide [1–4]. To meet the demands of a growing population, wheat improvement has prioritized the development of high-yielding, high-quality, and disease-resistant cultivars. However, both selection and fixation of favorable alleles and the general genome-wide bottleneck effect reduce genetic diversity during crop improvement, limiting crop resilience to diverse environmental conditions and potentially constraining future improvement [1]. For instance, increased yield potential is associated with increased sensitivity to abiotic stress [5]. As important reservoirs of genetic diversity, landraces can offer enhanced adaptability to a wider range of environmental conditions [6–8].

The trade-off between yield optimization and adaptive capacity of wheat is also epitomized by the Green Revolution. The introduction of *Rht* semi-dwarfing genes was a key driver of significant wheat yield increases. The favorable alleles *Rht-D1b* and *Rht-B1b* have been incorporated into modern cultivars [9–11]. However, *Rht-D1b* and *Rht-B1b* can be less effective in certain challenging environments. These alleles impair seedling emergence under deep-sowing practices in moisture-limited regions and reduce fertility in high-temperature environments [12, 13]. This varied performance of the same genotype in different environments illustrates the concept of phenotypic plasticity [14–17].

**Fig. 1:**
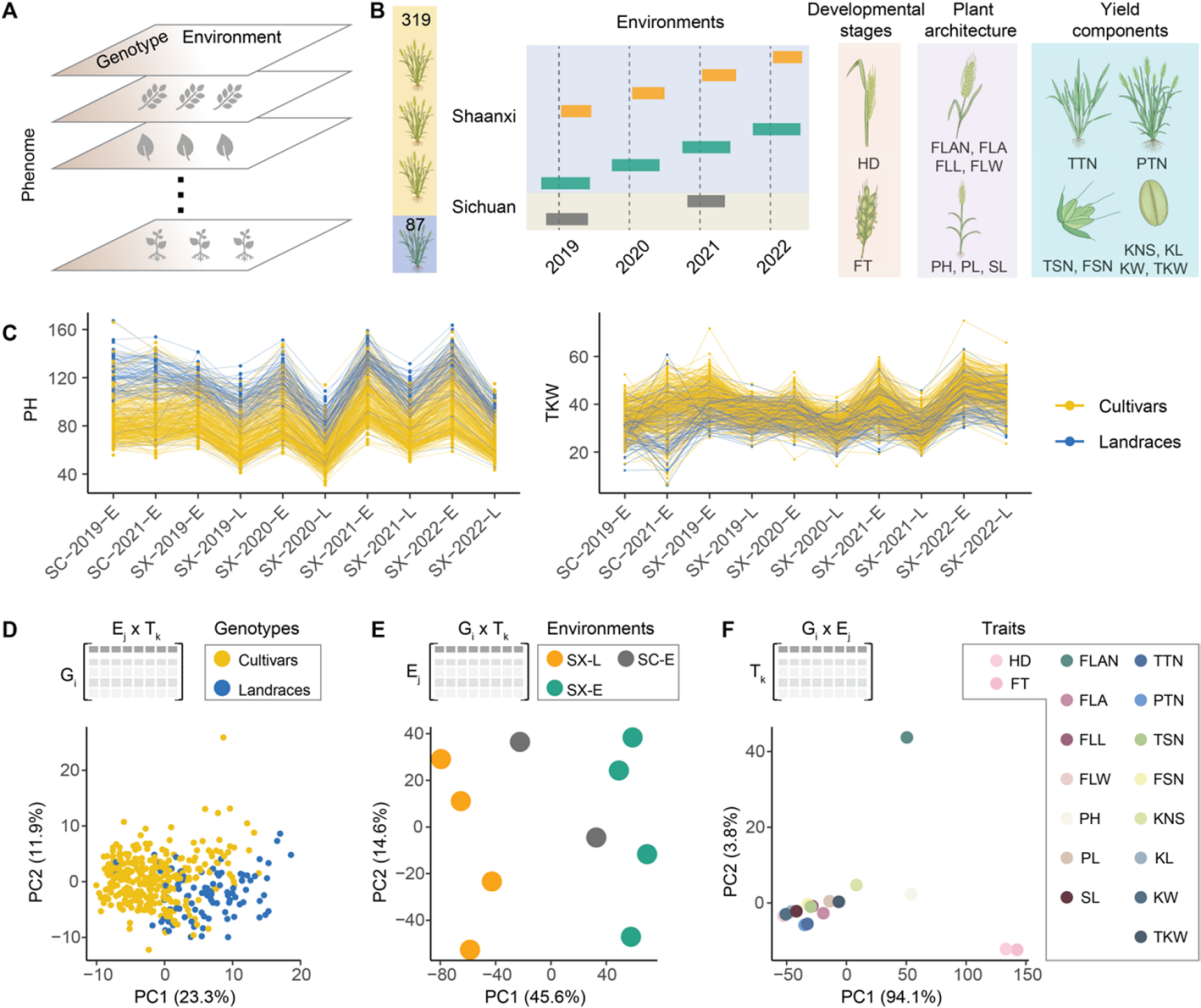
Latent patterns embedded in wheat phenotypic space. **A** A conceptual diagram illustrating phenome across traits, genotypes, and environments. **B** Key constituents of this large-scale multi-environment trial. The diverse wheat population consisting of landraces (blue) to cultivars (yellow) contains an evolutionary time dimension. Combinations of locations, years, and planting dates produce 10 unique environments. Three phenotypic categories account for 17 measured traits. **C** Fluctuations of plant height (PH) and thousand kernel weight (TKW) across environments. Each line connects observations of the same accession. **D**-**F** Major patterns embedded in the phenotypic space attribute to differences in genotypes (**D**), environments (**E**), and traits (**F**). Insets in D-F illustrate dimensions of the reshaped matrices from the 3D tensor for principal component analysis (PCA). PC1 represents principal component 1 and PC2 represents principal component 2

A systematic evaluation of how phenotypic plasticity was altered during crop improvement should improve our understanding of crop adaptability and resilience. Conducting a multi-environment trial in natural fields with a genetic population is a prerequisite to exploring phenotypic plasticity (Fig. 1A). Evidence from such experiments indicates that environment is as critical as genetics in explaining overall phenotypic variation [18–21]. Various approaches have been developed to study phenotypic plasticity from different perspectives [22–26]. Partitioning genetic and environmental contributions to phenotypic plasticity at a finer resolution, *i.e.*, major genetic and environmental determinants, could greatly facilitate a better understanding [27]. Integrations of genetic and environmental determinants have been applied to studies with different genetic backgrounds in different crops [28–32]. At the same time, due to the challenge of phenotyping across multiple environments, phenotypic plasticity studies using large genetic populations are typically limited to a few traits; therefore, how the improvement of a trait impacts the performance of other traits and their phenotypic plasticity remains largely unexplored. Furthermore, the genetic populations used in previous studies lacked the evolutionary time element; therefore, how phenotypic plasticity has changed during crop improvement has not been systematically explored.

In this study, we reported a comprehensive multi-environment field trial with a diverse wheat (*Triticum aestivum* L.) population consisting of landraces and cultivars. An array of 17 traits measured from 10 environments depicted the complexity of phenotypic variation at the genome, envirome, and phenome scales. We showed that two reaction-norm parameters (intercept and slope) based on the environmental indices identified by CERIS adequately captured trait variation across environments. We observed a broader spectrum of pleiotropic effects on phenotypic plasticity for *Rht-D1* than *Rht-B1*. The large number of traits and the evolutionary time element further enabled an overview of modes in altering phenotypic plasticity during wheat improvement.

## Results

### Phenome-wide wheat phenotypic plasticity

To systematically evaluate wheat phenotypic plasticity, we conducted a large-scale field experiment with a diverse population that consisted of 87 landraces and 319 cultivars (Fig. 1B; Additional file 1: Table S1) [33, 34]. All accessions were planted in 10 environments (unique combinations of location, year, and planting date) with three replications (Fig. 1B; Additional file 1: Table S2). A total of 17 traits, including two related to developmental stages, seven to plant architecture, and eight to yield components, were measured (nine traits were not measured in the SC-2021-E environment) (Additional file 1: Table S3). With up to nine individual plants evaluated within each replication, we collected 436,796 raw measurements (Additional file 1: Table S4). Within each environment, the high correlations (an average of 0.865) between replications and the high broad sense heritability estimates (an average of 0.897) demonstrated the quality of these measurements (Additional file 2: Fig. S1A). With the average value across replications within each environment, 65,366 observations in a three-dimensional (genotype × environment × trait) tensor represented a comprehensive phenotypic space for wheat (Additional file 1: Table S5).

We observed complex patterns in this phenotypic space from the genotypic, environmental, and trait perspectives (Fig. 1C; Additional file 2: Fig. S2). For instance, rankings of plant height (PH) among 406 accessions, which were largely consistent across environments (landraces generally being taller than cultivars), had an average *Spearman* correlation coefficient (*ρ*) of 0.821 with a standard deviation (SD) of 0.125 across 82,215 pairwise comparisons. In contrast, the mean and SD of *ρ* was 0.579 and 0.225 for thousand kernel weight (TKW), respectively (Fig. 1C). We partitioned the overall phenotypic variation into the aggregated genotype term, environment term, and genotype-by-environment interaction (G × E) term through an analysis of variance (Additional file 2: Fig. S1B). The environment term contributed the most to the overall variance for developmental stage traits (97.599% and 98.215%), followed by yield component traits (43.183% ± 22.116%, mean ± SD) and plant architecture traits (29.448% ± 9.593%). In contrast, the genotype term explained the highest proportion for plant architecture traits (38.320% ± 14.254%), followed by yield components (16.526% ± 7.769%), and developmental stage traits (1.195% and 0.910%). The heritability estimates across all environments were high for all 17 traits (Additional file 2: Fig. S1C). These results highlighted the distinct contributions of genotype, environment, and their interaction across different trait categories.

We investigated latent patterns embedded in this 3D phenotypic space through principal component analysis (PCA). As PCA transforms a 2D (*n* × *m*) matrix into an *n* × *L* [*L* = min(*n*, *m*)] space, where each *L* dimension encompasses contributions from all *m* variables, three sets of PCA were conducted by re-organizing the 3D phenotypic tensor into three different 2D matrices (Fig. 1D-1F). The first two principal components (PC1 and PC2) obtained from the 2D matrix with the dimension of *n* = 406 accessions and *m* = 170 (environments × traits) explained 23.3% and 11.9% of the total variance, respectively. Within this space, a general separation between landraces and cultivars was noticeable (Fig. 1D). From the 2D matrix of *n* = 10 environments and *m* = 6902 (accessions × traits), PC1 explained 45.6% of total variance and clearly separated early or late planting environments, while PC2 explained 14% of total variance and separated the four years, suggesting the timing of planting contributed more to the overall phenotypic variance than year (Fig. 1E). From the 2D matrix of *n* = 17 traits and *m* = 4060 (accessions × environments), PC1 explained 94.15% of the variance and mainly separated two developmental stage traits from the other traits (Fig. 1F).

### Enviromic dissection of wheat phenotypic plasticity

**Fig. 2:**
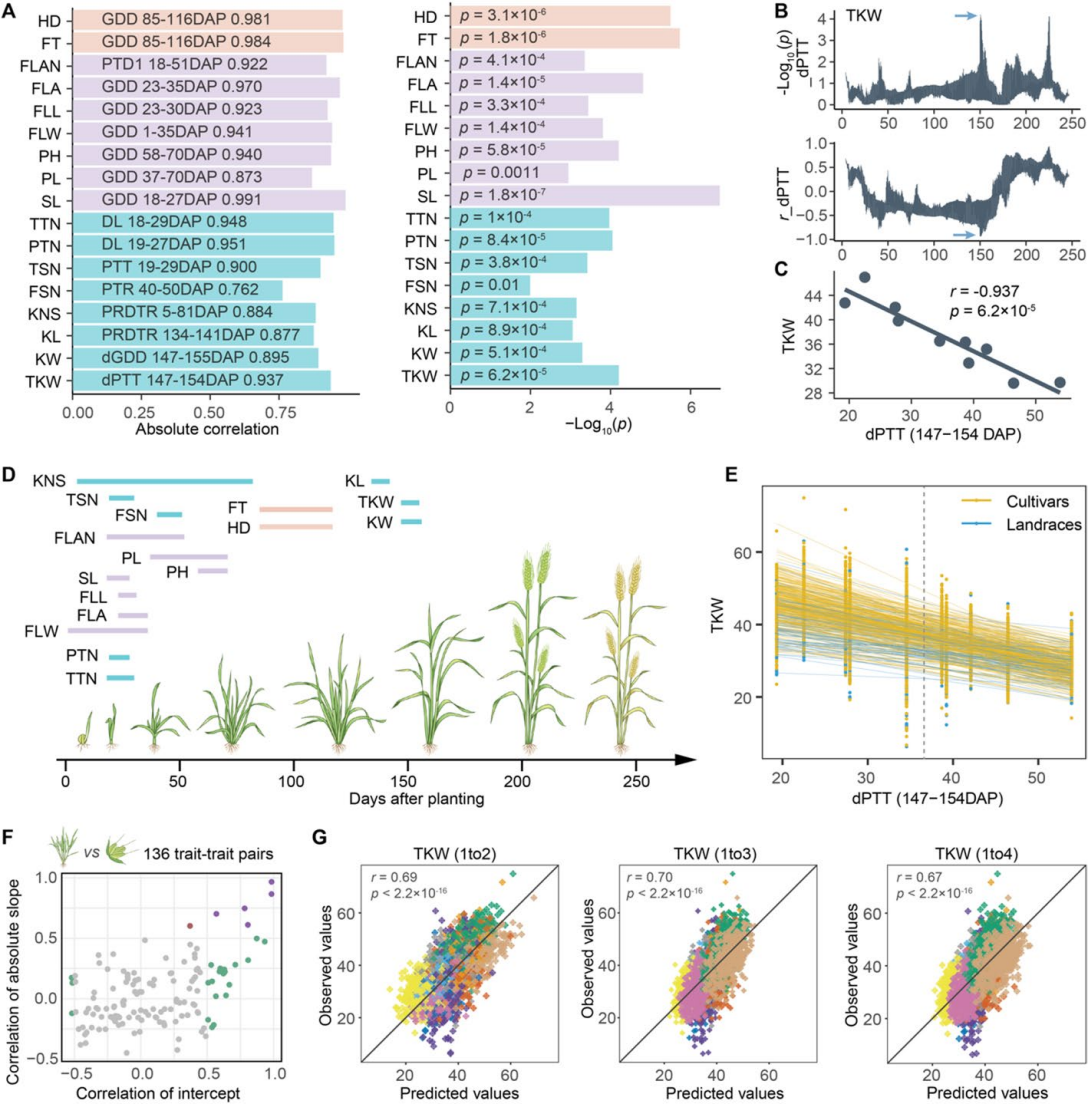
Modelling phenotypic plasticity with CERIS identified environmental index. **A** Correlations (in absolute value) and *p*-values between environmental mean and environmental index for each of 17 traits. **B** Identification of dPTT_147-154_ as the environmental index for thousand kernel weight (TKW). **C** The strong correlation between dPTT_147-154_ and environmental mean of TKW. Each dot represents one environment. **D** Growth periods associated with environmental indices for 17 traits. **E** Modelling TKW phenotypic variation of each accession based on dPTT_147-154_. The dashed vertical line marks the average value of the environmental index from which intercept was calculated for each accession. **F** Relationships of intercept and absolute slope among 136 trait-trait pairs. The x-axis represents the correlation of intercept of a pair of traits, while the y-axis represents the correlation of slope of the same pair of traits. Each dot denotes one pair of traits and the ones with an absolute correlation greater than 0.5 are color-coded. **G** Predictions of TKW under three performance prediction scenarios: tested genotypes in untested environments, untested genotypes in tested environments, and untested genotypes in untested environments. Each dot represents one accession and is color-coded by environment

To model the varied phenotypic values among environments, we applied CERIS to identify an environmental index that was strongly correlated with environmental mean, the average trait value of all accessions within each environment [29, 30]. CERIS not only considers weather factors, such as temperature or photoperiod, but also growth periods (measured as days after planting) of these factors. Of 17 identified environmental indices, 70% had an absolute *Pearson* correlation coefficient (|*r*|) greater than 0.900 (Fig. 2A). For example, differential photothermal time (dPTT) from 147 to 154 days after planting was the best choice to explain the variation in TKW across environments (*r* = -0.937, *p* = 6.2 × 10^–5^) (Fig. 2B, C). On average, developmental stage traits had the highest correlations (0.981 and 0.984, in absolute values) between environmental mean and environmental index, followed by plant architecture traits (0.937 ± 0.038), and yield component traits (0.884 ± 0.059) (Fig. 2A; Additional file 2: Fig. S3). Among 14 tested weather factors, Growing Degree Days (GDD), which measures the daily average temperature, contributed most to the identified environmental indices (chosen for eight out of 17 traits) (Fig. 2A; Additional file 2: Fig. S3). Most of the growth periods contributing to environmental indices identified by CERIS were before the transition from vegetative to reproductive stage, except for three kernel traits (Fig. 2D).

Identification of environmental indices allowed modelling of trait value fluctuations across environments for each wheat accession using a linear regression model (Fig. 2E; Additional file 2: Fig. S4). Each regression model had two parameters: intercept representing a genotype’s expected performance and slope capturing the level of sensitivity of a genotype to changes in environmental conditions. The sign of slope might vary across traits but is typically consistent among accessions within the same trait (Additional file 2: Fig. S4A). Higher absolute values of slope indicate higher sensitivity. These two reaction-norm parameters jointly describe the properties of each wheat accession in responding to environmental conditions, or phenotypic plasticity [35].

To investigate the relationship of phenotypic plasticity among traits, we calculated the pairwise correlations of the two reaction-norm parameters, intercept and slope (in absolute value), across the 406 accessions for all 136 possible pairs from 17 traits (Fig. 2F; Additional file 2: Fig. S5A, S6A). Of the 136 total pairwise comparisons, 26 pairs of intercept and 6 pairs of slope had significant (*p* < 0.05) and strong correlations (|*r*|> 0.5). We found both parameters were highly correlated for 5 pairs of traits, such as total tiller number (TTN) and productive tiller number (PTN) (*r* = 0.984 for intercept and 0.967 for slope) (Additional file 2: Fig. S5A, S6A). 21 pairs of traits had a strong (|*r*|> 0.5) correlation of intercept but a weak correlation (|*r*|< 0.5) of slope, such as PH and peduncle length (PL) (*r* = 0.933 for intercept but 0.473 for slope) (Fig. 2F; Additional file 2: Fig. S5A, S6A). One pair, spike length (SL) and total spikelet number (TSN), displayed a strong correlation of slope (*r* = 0.603) but weak correlation of intercept (*r* = 0.370) (Fig. 2F; Additional file 2: Fig. S5A, S6A). These findings revealed diverse relationships among traits, suggesting their underlying connections in terms of how plants allocated resources in response to environmental conditions. Increasing yield is a top priority of crop improvement. Among eight yield component traits, three (PTN, kernel number per spike (KNS), and TKW) directly contribute to yield per plant. Intercept of these three traits were significantly but negatively correlated, reflecting the trade-offs and competition among these yield components (Additional file 2: Fig. S5B). Meanwhile, among three pairs of slope, only the pair between PTN and TKW was significantly and negatively correlated, suggesting accessions that were highly sensitive to the environmental conditions impacting TKW were generally less sensitive to the environmental conditions impacting PTN (Additional file 2: Fig. S6B).

A benefit of identifying an environmental index is to predict crop performance under new environments [29, 30]. We tested three prediction scenarios: the 1to2 scenario of predicting performance of tested genotypes in untested environments, the 1to3 scenario of predicting performance of untested genotypes in tested environments, and the 1to4 scenario of predicting performance of untested genotypes in untested environments (Fig. 2G; Additional file 1: Fig. S7-9). Consistent with previous reports, the prediction accuracies were the highest for the 1to2 prediction scenario, with values ranging from 0.571 to 0.972 across 17 traits. The prediction accuracies between the 1to3 and 1to4 scenarios were comparable, even though the 1to4 scenario had an additional uncertainty of environment (Additional file 2: Fig. S10). The consistently high prediction accuracies and the ratios between predicted and observed values further supported that two reaction-norm parameters adequately captured phenotypic variation. We also showed that incorporating environmental factors substantially improved prediction accuracies (Additional file 2: Fig. S11).

### Genetic dissection of wheat phenotypic plasticity

**Fig. 3:**
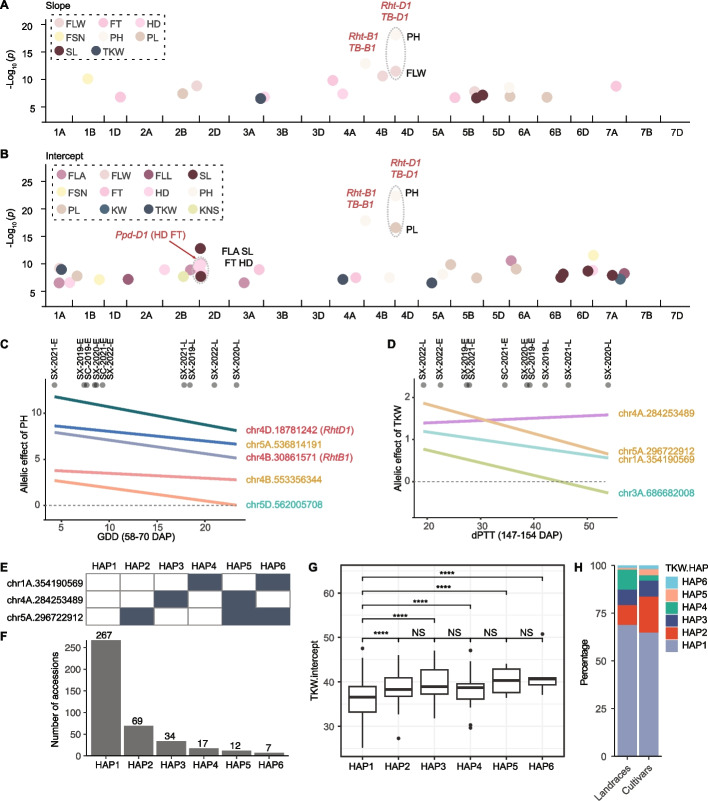
Genetic architecture of wheat phenotypic plasticity. **A**, **B** Manhattan plots show the genome-wide association results for slope (**A**) and intercept (**B**) for 17 traits. Each dot represents a significant single nucleotide polymorphism (SNP). **C**, **D** Modelling allelic effects of significant SNPs related to plant height (PH) (**C**) and thousand kernel weight (TKW) (**D**) phenotypic plasticity based on the environmental index. Each line represents a distinct SNP, with SNP names in red indicating those associated with both intercept and slope, teal for slope only, and orange for intercept only. **E** Six observed haplotypes across three SNPs associated with TKW intercept. **F** Frequencies of these six haplotypes. **G** Variation of TKW intercept values across haplotypes, with significant difference indicated by ∗ ∗ ∗ ∗ (*p* < 0.0001) and “NS” for non-significant difference. **H** HAP2 is enriched in cultivars during wheat improvement

To explore genetic architecture underlying phenotypic plasticity, we conducted GWAS to identify loci associated with variation in the two reaction-norm parameters. Among 157,050 SNPs discovered through sequencing mRNAs extracted from roots of the 406 wheat accessions (Additional file 2: Fig. S12), 22 loci significantly associated with slope variation for 8 traits and 39 loci associated with intercept variation for 12 traits were identified (Fig. 3A, B; Additional file 1: Table S6). Several well characterized genes were tagged by significant SNPs (Additional file 1: Table S7). *Ppd-D1*, one of the key genes regulating photoperiod responses of wheat [36], was tagged by a SNP at position 32,970,833 on chr2D. This SNP was significantly associated with intercept for both heading date (HD) and flowering time (FT) (Fig. 3B). Two SNPs, located at 18,781,242 on chr4D and 30,861,571 on chr4B were significantly associated with both intercept and slope for PH (Fig. 3A, B). These two SNPs, each introducing a premature stop codon, corresponded to the previously reported functional sites of *Rht-D1b* and *Rht-B1b*, respectively (Additional file 2: Fig. S13A, S13B). As expected, alleles associated with early flowering and semi-dwarf phenotypes had higher frequencies in cultivars than in landraces (Additional file 2: Fig. S13C).

Varied additive effects of a SNP between a pair of environments can be classified into three possible categories: antagonistic pleiotropy, conditional neutrality, or differential sensitivity [37]. Among nine SNPs significantly associated with the reaction-norm parameters of PH or TKW, seven had genetic effects that varied in magnitude. In other words, all pairwise additive effects among tested environments belonged to the differential sensitivity category (Fig. 3C, D). Two SNPs (chr5D.562005708 associated with slope of PH and chr3A.686682008 associated with slope of TKW) had no significant additive effects in SX-2020-L, suggesting the possibility of conditional neutrality from certain pairs of environments. Furthermore, the SNP of chr3A.686682008 could be in the antagonistic pleiotropy category given the reversed additive effects on TKW in certain conditions. These observations were in agreement with previous studies that differential sensitivity is the dominant type of effect dynamic [27, 29, 37].

GWAS identified three significant loci associated with intercept of TKW. Of eight possible haplotypes across three loci, six were observed with varied frequencies within the 406 wheat accessions (Fig. 3E, F). The predominant haplotype (HAP1, 65.8%) had the lowest average intercept (Fig. 3G). The proportion of the beneficial HAP2 (increasing intercept of TKW) was 1.8-fold higher in cultivars than in landraces, alluding to the potential selection of HAP2 to increase kernel weight during wheat improvement (Fig. 3H).

### Pleiotropic effects of *Rht-D1* and *Rht-B1* on wheat phenome-wide phenotypic plasticity

**Fig. 4:**
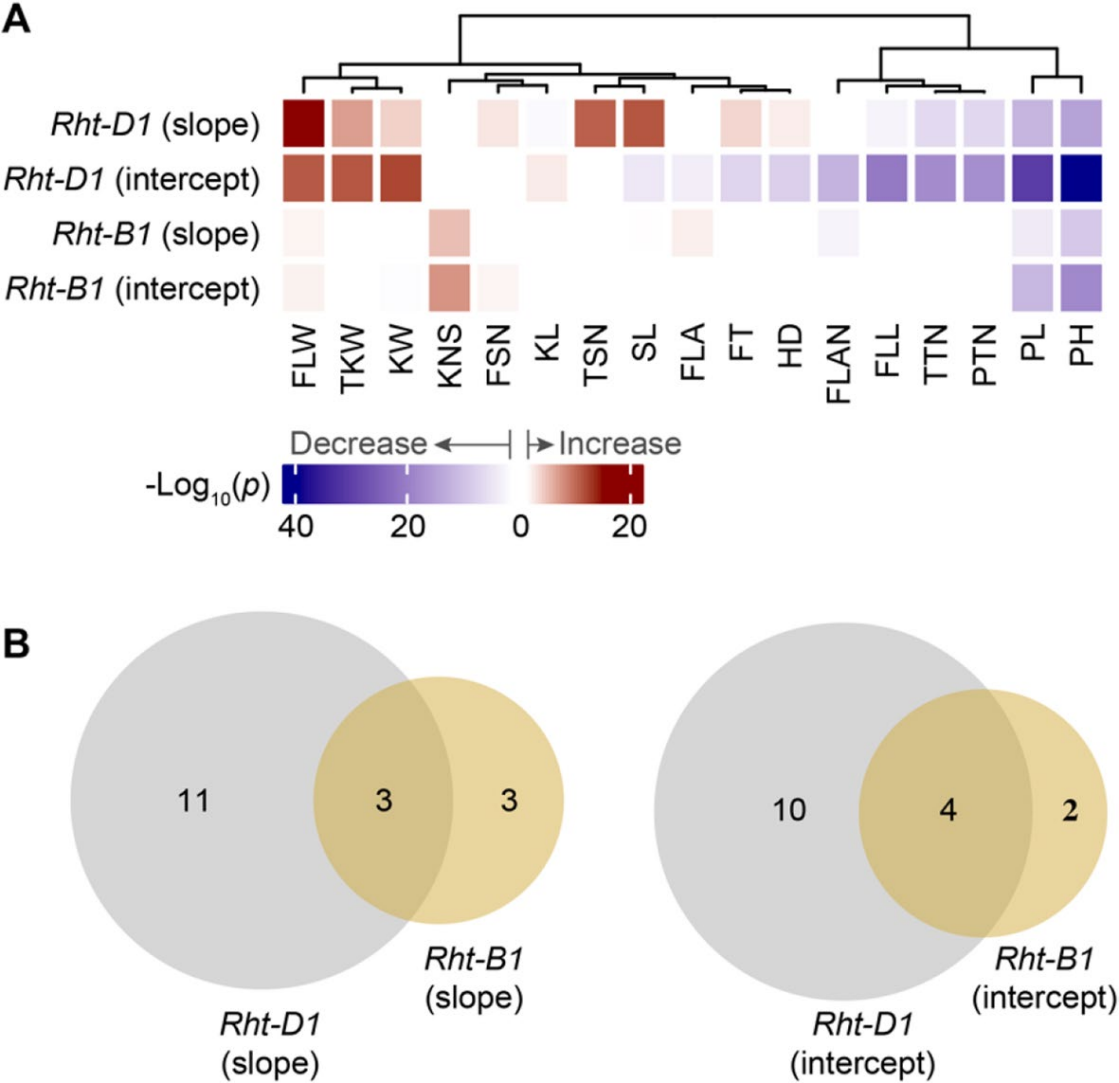
Pleiotropic effects of *Rht-D1* and *Rht-B1* on phenotypic plasticity across 17 traits. **A** Spectrum of significant effects of *Rht-D1* and *Rht-B1* on intercept and slope of 17 traits. The changing direction of the effect from the wildtype to the mutant is indicated by color (red for increase and blue for decrease) while the intensity indicates the level of significance. **B** *Rht-D1b* alters slope (left) and intercept (right) of more traits than *Rht-B1b*

Mutations of *Rht-D1* and *Rht-B1* have been widely selected to reduce plant height and enhance lodging resistance [38]. GWAS indicated that both *Rht-B1* and *Rht-D1* were significantly associated with the two reaction-norm parameters for PH. Conversely, only *Rht-D1* significantly contributed to variation in PL intercept and flag leaf width (FLW) slope, implying a different spectrum of pleiotropic effects of these two Green Revolution genes in altering wheat phenotypic plasticity (Fig. 3A, B). Because the functional polymorphisms of both genes were genotyped, we directly compared the intercept and slope of all evaluated traits between wild-type and mutant alleles of these two genes. In comparison to the corresponding wild-type allele, we observed a broader spectrum of *Rht-D1b* than *Rht-B1b* in altering phenotypic plasticity across multiple traits (Fig. 4; Additional file 2: Fig. S14, S 15). Interestingly, among 17 measured traits, *Rht-D1* affected the slope of 14 traits, except KNS, flag leaf angle (FLAN), and flag leaf area (FLA). In contrast, *Rht-B1* only altered slope for KNS, FLAN, FLA, and three other traits. Two genes jointly impacted the slope estimates of all evaluated traits. At the intercept level, *Rht-D1* significantly influenced 14 traits (except TSN, FSN, and KNS), while *Rht-B1* displayed a substantial impact only on PH, PL, and KNS. Among the three traits (PTN, KNS and TKW) contributing to yield per plant, *Rht-D1b* increased TKW, while *Rht-B1b* increased KNS (Fig. 4).

### Modes of phenotypic plasticity change during wheat improvement

To further understand the changes in phenotypic plasticity during wheat improvement, we compared intercept and slope (in absolute value) between landraces and cultivars (Additional file 2: Fig. S16). A higher intercept estimate suggests higher genetic potential, while a higher slope estimate suggests increased sensitivity to changes in environmental conditions. From landraces to cultivars, each parameter could have three outcomes: decreasing, constant, or increasing. Among 17 evaluated traits, cultivars had significantly lower intercept for ten traits, including two developmental stage traits, six (out of seven) plant architecture traits, and two yield component traits (TTN and PTN), while cultivars had higher intercept for five traits, of which four were yield component traits (Additional file 2: Fig. S16). This observation indicated that the increased yield potential of cultivars was associated with the suppressed values of traits contributing less directly to yield, a desired outcome of resource allocation from the perspective of wheat improvement. On the other hand, cultivars had significantly lower slope for six traits and higher slope for ten traits. An obvious pattern is, among ten traits with increased slope in cultivars, half were yield component traits (KW, TKW, KNS, FSN, and TSN). This finding indicates that wheat improvement has generally increased sensitivity to changes in environmental conditions, particularly for yield component traits (Additional file 2: Fig. S16).

**Fig. 5:**
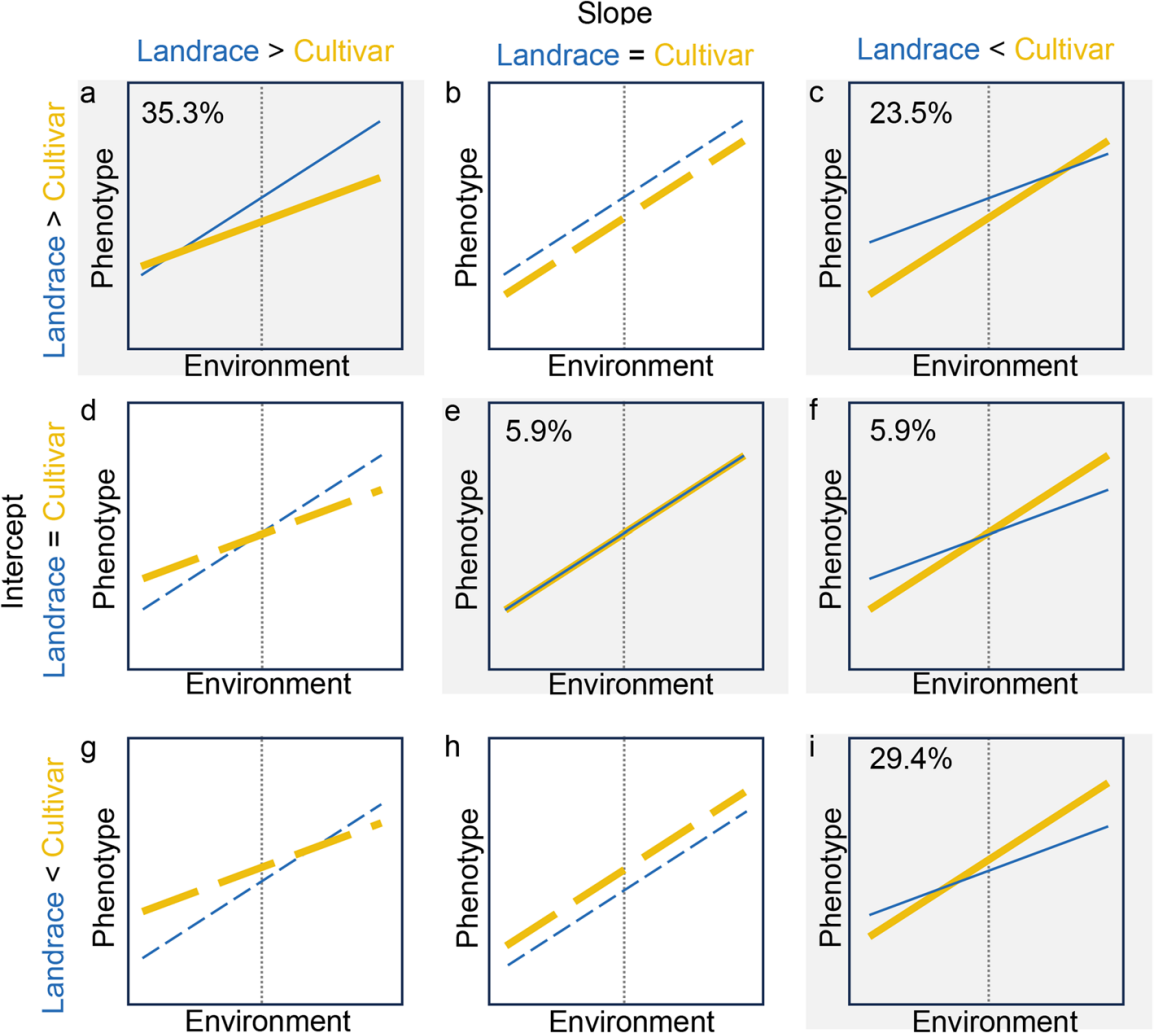
Modes of phenotypic plasticity change from landraces to cultivars. Among nine possible modes, five are observed in this study, with three as the dominant modes (indicated by the percentage). Dashed colored lines and white background denote unobserved modes. Dotted gray vertical lines mark the average value of the environmental index from which intercept is calculated

Considering the changes of intercept and slope simultaneously generates a total of nine (3 × 3) potential modes of phenotypic plasticity change (Fig. 5). Of these nine possible modes, we observed five modes within 17 evaluated wheat traits, with three (*a*, *c* and *i*) as predominant modes. Mode *a* of decreased intercept and decreased slope in cultivars is the most frequent, which included six traits, four plant architecture traits (FLL, FLAN, PL, and PH) and two yield component traits (PTN and TTN) (Additional file 2: Fig. S16A). Out of the five traits classified as Mode *i* of increased intercept and increased slope in cultivars, four (FSN, KNS, TKW, and KW) were yield component traits. Two developmental stage traits and two plant architecture traits experienced decreased intercept but increased slope, or Mode *c*. Overall, wheat improvement has modified 23.5% of traits by decreasing intercept but increasing slope, while 64.7% of traits were modified in the same direction. Furthermore, plant architecture traits were modified towards lower intercept and sensitivity, while yield component traits were modified towards greater intercept and sensitivity (Fig. 5; Additional file 2: Fig. S16). These results provide a comprehensive view of the shifts in phenotypic plasticity during wheat improvement.

## Discussion

Phenotypic plasticity emerges from the interaction between genotypic and environmental conditions [14]. Elucidating this interaction by incorporating environmental components is a long standing question [37, 39]. Previous studies on understanding crop phenotypic plasticity have predominantly explored a limited number of traits [29, 30]. The large set of traits we measured from multiple environments enabled not only evaluation of overlooked relationships among traits [40–42], but also how changes in one trait may impact other traits under varying environmental conditions. Correlations of the reaction-norm parameters among traits and genetic architectures supported complex patterns of phenome-wide phenotypic plasticity. Three yield component traits exhibited antagonistic relationships at the intercept level but only one pair (TKW and PTN) had a significant negative correlation at the slope level. This observation suggests the possibility of selecting for varieties with higher TKW that maintain stable PTN under specific environments. For the two globally utilized Green Revolution genes (*Rht-D1* and *Rht-B1*), their pleiotropic effects on phenome-wide phenotypic plasticity differed. Meanwhile, the mutations in both genes collectively altered two reaction-norm parameters of all evaluated traits, except intercept for one trait. Notably, *Rht-D1b* increased TKW and *Rht-B1b* enhanced KNS; however, both alleles also elevated environmental sensitivity. These results indicate the importance of considering genomic and environmental interactions in breeding strategies for enhancing genetic potential and environmental sensitivity.

Our study introduced the evolutionary time dimension into exploring phenotypic plasticity. Binary classification of the changing directions of the two reaction-norm phenotypic plasticity parameters along the evolutionary time dimension leads to nine potential modes, which provides a structured overview of how breeding and other evolutionary scenes might reshape the ability of a species to respond to environmental conditions. The majority of the studied wheat traits, especially the plant architecture and yield component traits, were altered as intercept and slope moving in the same direction during wheat improvement [5]. Meanwhile, a quarter of the traits were reshaped with intercept and slope moving in opposite directions. The implications of these findings extend beyond theoretical modelling to practical applications. For example, the increased slope in cultivars observed in 50% of traits highlights a trend toward greater environmental sensitivity, which may be beneficial under optimal conditions, but may pose risks in stress-prone environments.

While this study provides significant insights into phenome-wide phenotypic plasticity change during crop improvement, several limitations should be acknowledged. First, we modelled the phenotypic fluctuations of each genotype across environments with a linear regression. Future studies might explore advanced machine learning and other artificial intelligence models [43]. Second, due to the large genome size of wheat, we leveraged a cost-effective RNA-seq strategy for SNP identification. Incorporating whole-genome sequencing or high-density genotyping platforms in future studies would improve the resolution of genotype–phenotype associations. Finally, while we identified significant patterns of phenotypic plasticity across traits, the underlying molecular mechanisms driving these changes remain to be investigated [44].

## Conclusions

Exploiting phenotypic plasticity is of great importance in improving crops. Through measuring a suite of 17 traits from both wheat landraces and cultivars across environments, our comprehensive analyses revealed a global view of phenotypic plasticity change during the improvement of wheat. Traits directly contributed to yield were generally altered as higher intercept and sensitivity, while plant architecture traits were altered towards lower intercept and sensitivity.

## Methods

### Plant materials, genotyping and phenotyping

A total of 406 bread wheat accessions including 87 landraces and 319 cultivars were collected across the globe (Additional file 1: Table S1) [33]. These accessions have short vernalization period requirements and adequate cold tolerance, and therefore, can be planted in both January and October to finish the life cycle from seeds to seeds.

RNA samples from the 406 accessions were collected from roots and sequenced on the Illumina HiSeq X Ten platform, with reads aligned to the bread wheat genome (IWGSC RefSeq v1.0) using STAR [45]. Uniquely mapped reads were selected for SNP calling. A modified GATK pipeline [46] identified high-quality SNPs with strict filtering criteria (https://github.com/biozhp/Population_RNA-seq). Missing genotypes (< 0.8 missing rate) were imputed using Beagle (v5.4) [47], ensuring > 99% accuracy in the final SNP dataset [33].

The 406 wheat accessions were planted in ten environments over four years with early or late planting (E or L) in two locations (Yangling, Shaanxi and Chongzhou, Sichuan) (Additional file 1: Table S2). The design of two plantings served the dual purposes of evaluating thermotolerance of wheat [48] and creating a wider environmental mean range to evaluate phenotypic plasticity [49]. A randomized complete block design with each accession planted in three replications was implemented in each environment. Each accession was sown in rows 1 m in length with a row spacing of 20 cm and 10 seeds per row. Field management was consistent with local practices for wheat production to control weeds and diseases. The disease/pest regimen, applied in April, included 20 mL/ha Bayer Sphere® (10% trifloxystrobin + 20% tebuconazole), 40 mL/ha bifenthrin, and 4 g/ha imidacloprid.

Seventeen traits were measured, including developmental stage traits: heading date (HD) and flowering time (FT); plant architecture traits: plant height (PH), peduncle length (PL), flag leaf angle (FLAN), flag leaf area (FLA), flag leaf length (FLL), flag leaf width (FLW) and spike length (SL); and yield component traits: total tiller number (TTN), productive tiller number (PTN), total spikelet number (TSN), fertile spikelet number (FSN), kernel number per spike (KNS), kernel length (KL), kernel width (KW), and thousand kernel weight (TKW) (Additional file 1: Table S3). The phenotyping protocol is documented in Additional file 1: Table S3. Eight of 17 traits (FSN, KL, KNS, KW, PH, PL, TKW, and TSN) were measured in all environments and the remaining nine traits (FLA, FLAN, FLL, FLW, FT, HD, PTN, SL, and TTN) were measured in nine environments (all except SC-2021-E) (Additional file 1: Table S2). Three to nine plants per replication were measured (Additional file 1: Table S4). After calculating the average value of replicates within each environment, missing values were imputed using the *missForest* package in R (Additional file 1: Table S5).

### Analysis of variance, heritability, and PCA

For each trait, the *lmer* function from the *lme4* package in R was used to estimate contributions from genotype, environment, and their interaction to the overall trait variation. The broad-sense heritability in each environment was calculated using the following equation: *H*^*2*^ = σ_g_^2^ / [σ_g_^2^ + (σ_e_^2^ / r)], where σ_g_^2^ represents genotypic variance, σ_e_^2^ represents residual variance for each environment, and r represents the number of replicates. Then the heritability across the environments was calculated using the equation: *H*^*2*^ = σ_g_^2^ / [σ_g_^2^ + (σ_ge_^2^ / e) + (σ_e_^2^ / re)], where σ_g_^2^, σ_ge_^2^, and σ_e_^2^ represent genotypic, genotype by environment, and residual variance, respectively, r represents the number of replicates, and e represents the number of environments. After reshaping the original 3D phenotypic tensor (genotype × environment × trait) into a 2D matrix, the *prcomp* function in R was called to conduct principal component analysis.

### CERIS-JGRA

Five primary environmental factors, including day length, the maximum of daily temperature (T_max_), the minimum of daily temperature (T_min_), precipitation, and relative humidity, were obtained from NASA POWER (https://power.larc.nasa.gov/data-access-viewer/). From these, 14 environmental parameters, including DL (Day Length), GDD (Growing Degree Days), dGDD (Differential Growing Degree Days), DTR (Diurnal Temperature Range), PTT (Photothermal Time), dPTT (Differential Photothermal Time), PTR (Photothermal Ratio), PTD1 (Photothermal Diurnal Range), PTD2 (Photothermal Diurnal Ratio), TSR (Temperature Square Ratio), MMR (Minimum-to-Maximum Ratio), PR (Precipitation), RH (Relative Humidity), and PRDTR (Precipitation-to-Diurnal Temperature Range), were calculated (Additional file 1: Table S8).

The CERIS-JGRA framework (https://github.com/jmyu/CERIS_JGRA) was used to model phenotypic variation. The first stage of this framework is to identify an environmental index to quantify the environmental conditions, which is conducted by CERIS, a systematic, data-driven algorithm. The second stage is to model trait value variation across environments by regressing the trait values on the identified environmental index. As a result, multiple observations from each individual genotype can be captured by the two reaction-norm parameters. The whole process involved four steps: (1) Calculate the average values of any growth period from planting to harvesting (250 days after planting) for each of the 14 environmental parameters as potential environmental indices. (2) Calculate environmental mean for each environment for a given trait. (3) Estimate *Pearson* correlation coefficients between environmental mean and each of the potential environmental indices to identify the one with the strongest correlation within a biologically reasonable timeframe as the environmental index. (4) Regress the trait values across environments for each accession on the identified environmental index to obtain intercept and slope, the two reaction norm parameters. Most subsequent analyses were conducted using these two parameters.

Performance predictions of tested genotypes in untested environments (1to2) were conducted using a leave-one-environment-out cross-validation. For each untested environment, a linear regression model was trained with observations from all other remaining environments to estimate two reaction-norm parameters, then the value of the environmental index from the untested environment was fed into the model to predict the performance of each individual. The *Pearson* correlation coefficient between predicted and observed values were estimated within each environment and across all the environments for all traits. The ratio between predicted and observed values was also calculated for each accession within each environment.

Performance predictions of untested genotypes in tested environments (1to3) were conducted with five-fold cross-validation. The 406 accessions were randomly split into five subsets of approximately equal sample sizes. Accessions in each subset were designated as untested genotypes, while the remaining were tested genotypes. The two reaction-norm parameters were estimated based on the identified environmental index for each tested genotype. Treating each parameter as a trait, a genomic prediction model based on SNPs was trained using all tested genotypes through the *rrBLUP* package [50]. For an untested genotype, the two reaction-norm parameters were predicted through the corresponding genomic prediction model and were combined with environmental index values to predict the trait values in all environments. Each random split constituted one iteration with all five subsets being sequentially designated as untested genotypes. The mean values across 10 iterations were compared to the observed values to assess the prediction model.

Performance predictions of untested genotypes in untested environments (1to4) were conducted with a cross-validation combining leave-one-environment-out and five-fold on genotypes. 1to4 is a combination of 1to2 and 1to3. We used the *rrBLUP* package to predict the intercept and slope of an untested genotype, which was combined with environmental index to predict the phenotype of the untested environment. The mean value across 10 iterations was compared to the observed values to assess the prediction model.

### GWAS

GWAS were conducted to identify significant loci associated with the two reaction-norm parameters by the Blink model implemented in the *GAPIT* package [51]. PCA based on the SNPs was conducted by the functions encoded in *GAPIT*. The first three principal components (PCs) were included as fixed effects to adjust for population structure. Significant SNPs were annotated based on the reference genome of *Triticum aestivum* (IWGSC RefSeq v1.0), and candidate genes within a 1-Mb window around each significant locus were identified for further analysis.

To trace the additive effect dynamics for the original trait along the environmental dimension, the allele with the lower intercept value was set as the base allele. The allelic effect within each environment was estimated as the mean phenotypic value difference between the two alleles (the alternative minus the base allele). The allelic effects across all environments were then regressed on the environmental index by the *lm* function in R.

## Supplementary Information

Additional file 1: Tables S1–8.

Additional file 2: Fig. S1–16.

## Data Availability

The raw RNA-Seq data are available at Sequence Read Archive (https://www.ncbi.nlm.nih.gov/sra) under accession number PRJNA838764 [33, 52]. The genotypic data have been deposited in Zenodo (10.5281/zenodo.15805772) [33, 53]. Sample information and phenotypic data in this study are included in the supplementary tables.
