# Supplementary material for: Integrated phenomic and genomic analyses unveil modes of altered phenotypic plasticity during wheat improvement
Source: Genome Biol. 2025 Aug 28;26:256. doi: 10.1186/s13059-025-03740-1 (PMC12392622; doi:10.1186/s13059-025-03740-1)
Supplement: Supplementary file 2 — Additional file 2: Fig. S1–16. [file 13059_2025_3740_MOESM2_ESM.docx]

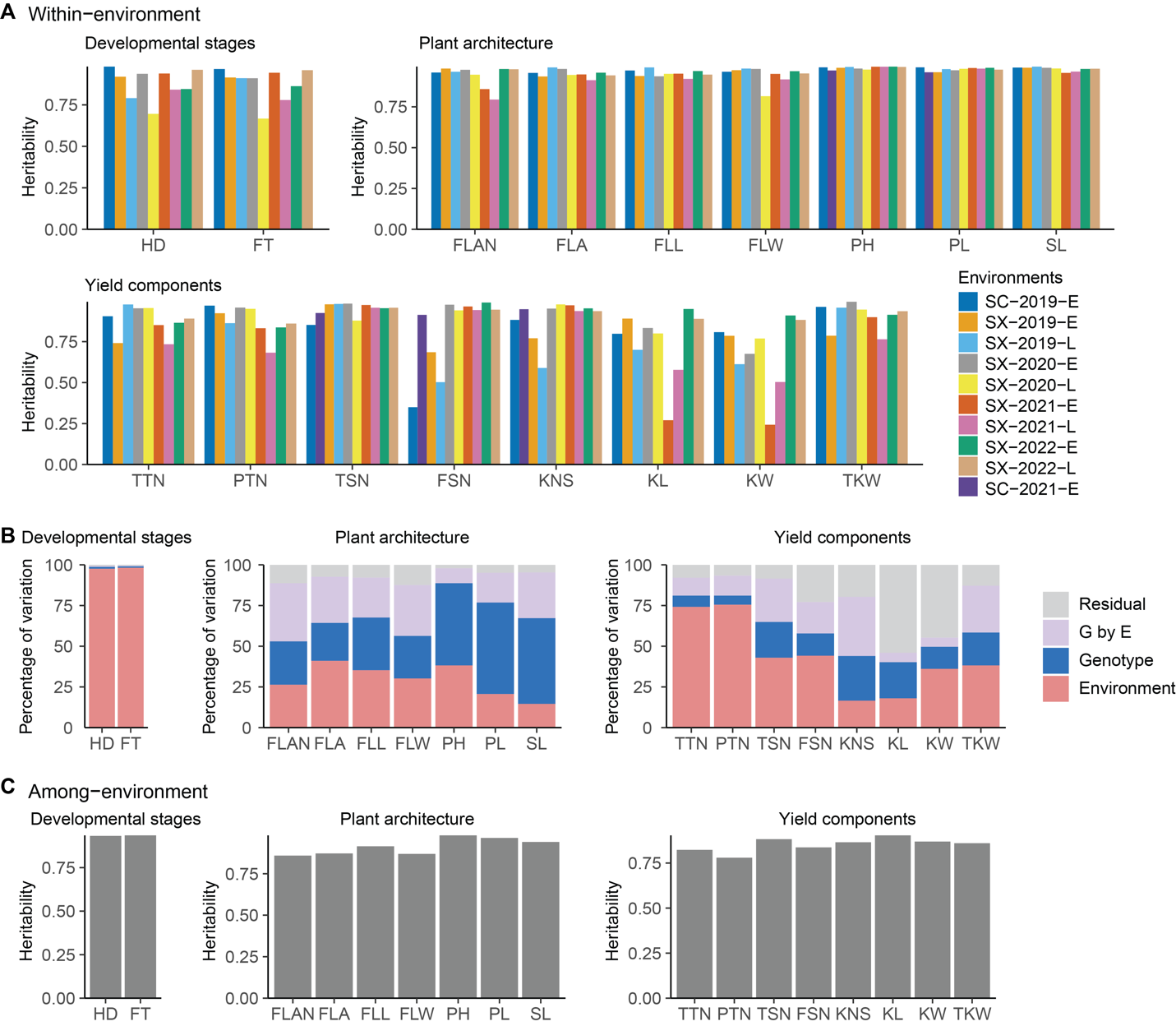


**Fig. S1. Partitioning phenotypic variance.** (A) The within-environment heritability. (B) Variance attributed to environment, genotype, G × E, and residual. (C) The among-environment heritability.


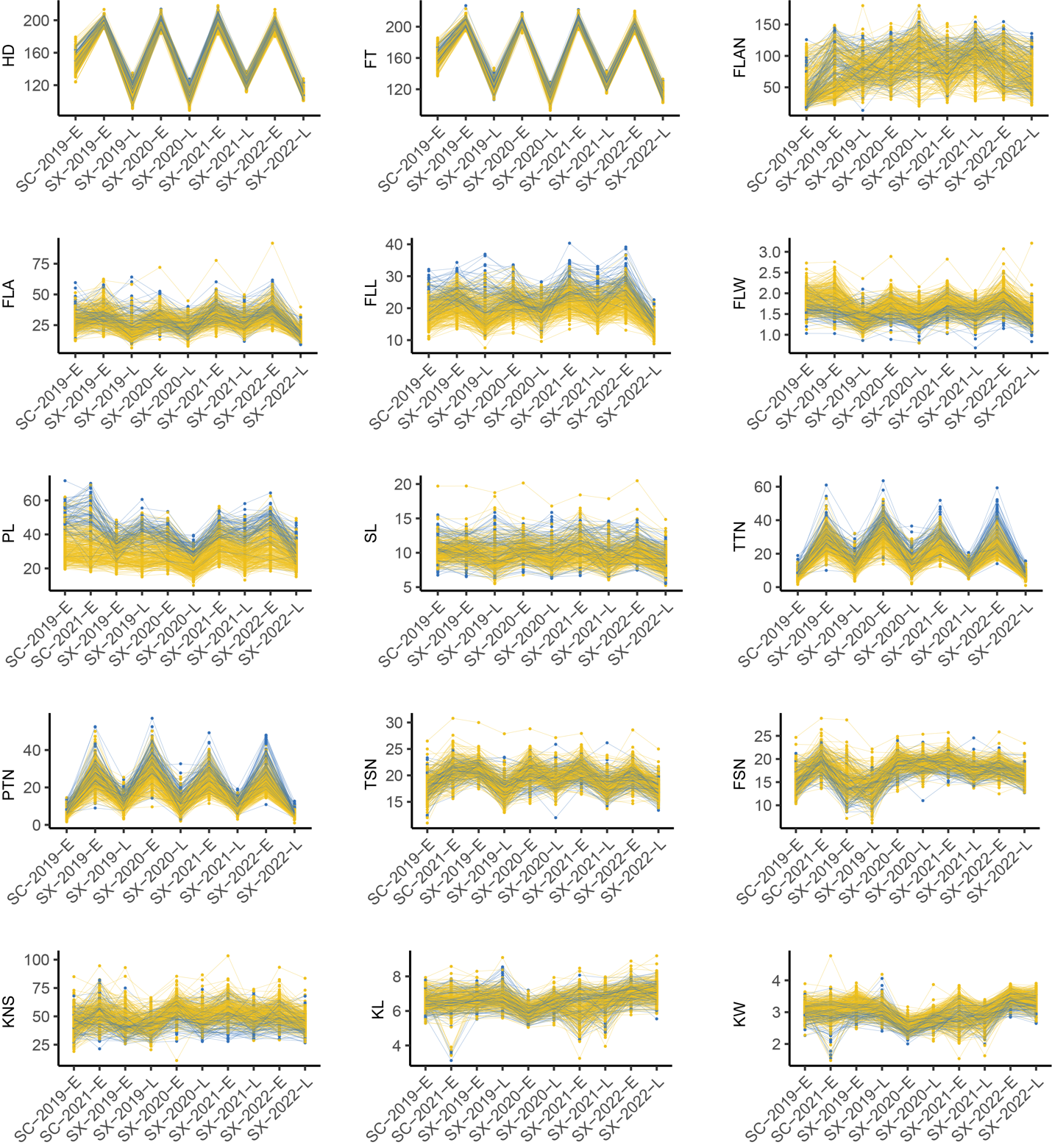


**Fig. S2. Trait fluctuations across environments.** Each line represents a distinct accession (yellow for cultivars and blue for landraces).


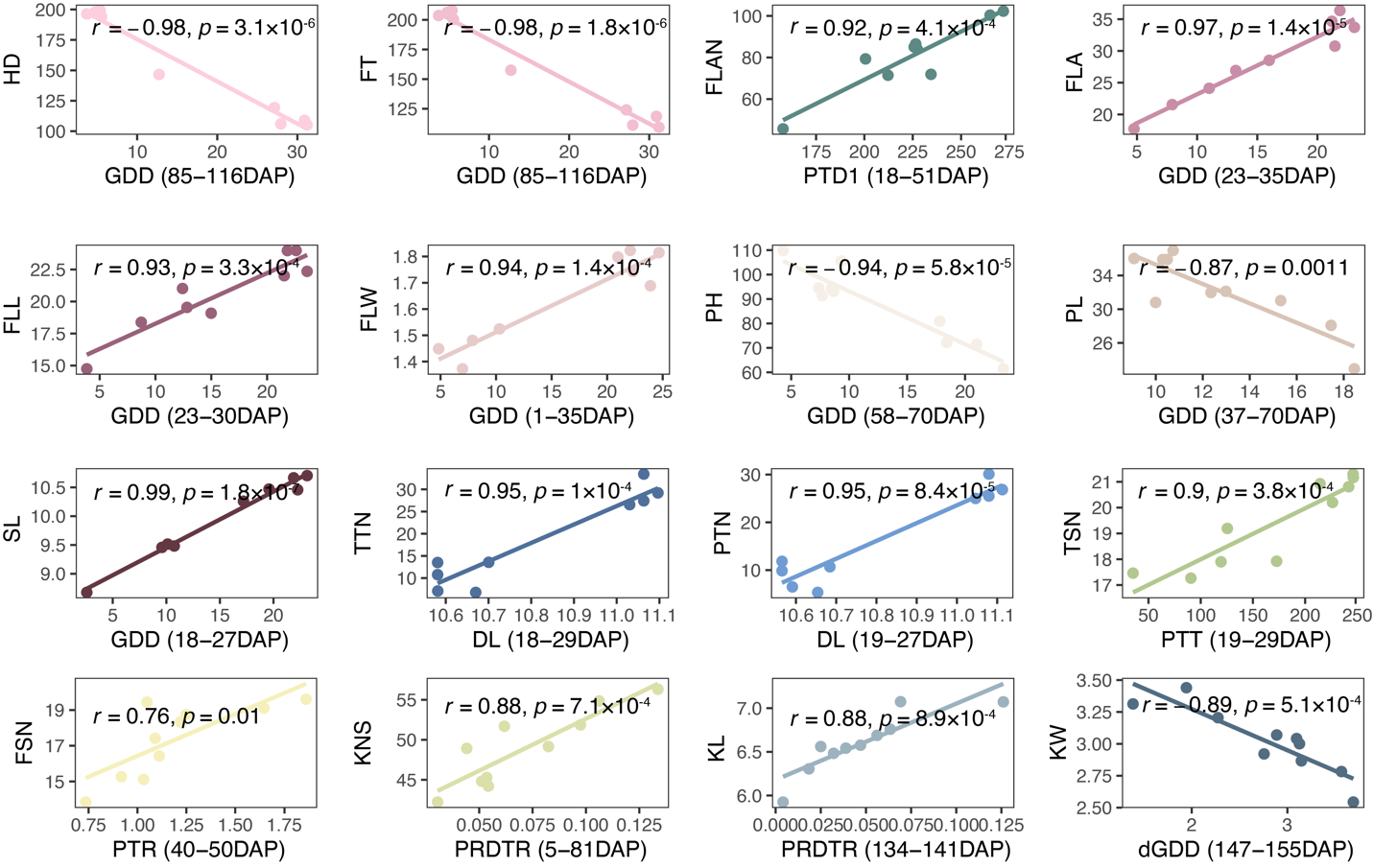


**Fig. S3. Correlations between environmental index and environmental mean.** Each dot represents one environment.


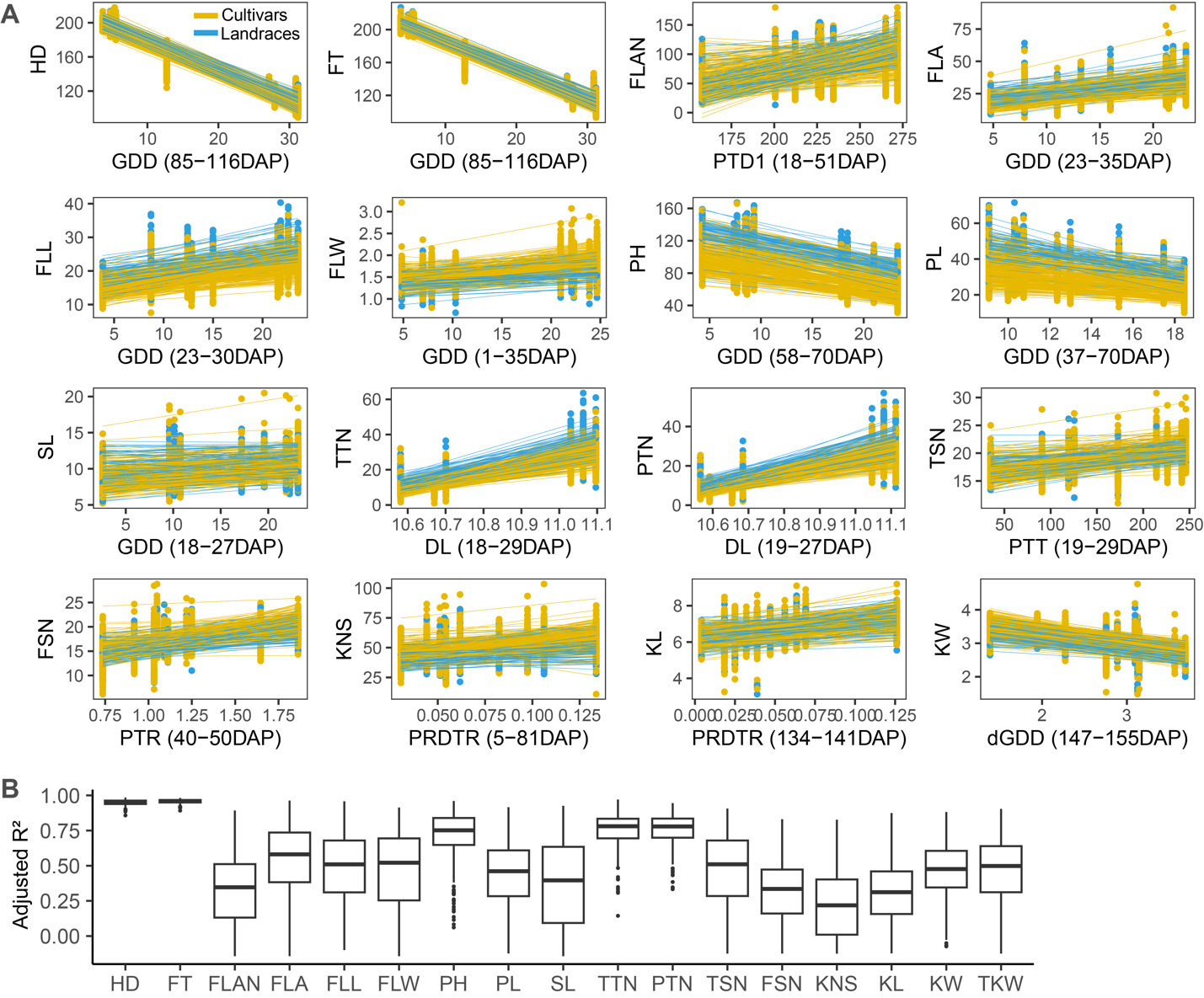


**Fig. S4. Performance dynamics of each accession modelled by environmental indices.** (A) Each dot represents the observed phenotypic value for each accession in each environment, and each line corresponds to the regression-fitted values for an individual accession. Yellow represents the lines from cultivars and blue represents the lines from landraces. (B) The distribution of the adjusted *R^2^* values across evaluated traits.


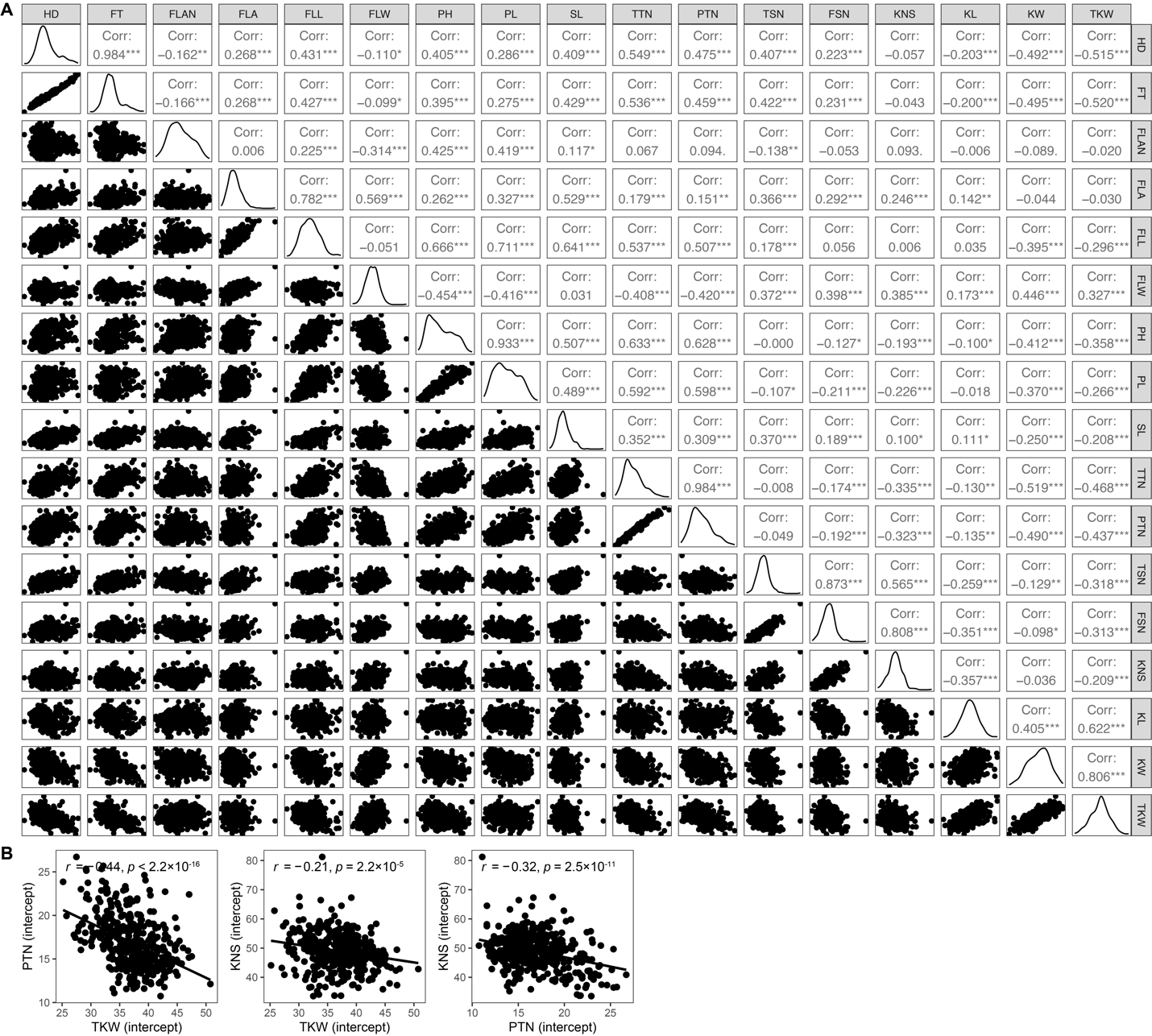


**Fig. S5. Pairwise correlation of intercept.** (A) Pairwise correlation of intercept for 17 traits. Each cell displays the *Pearson* correlation coefficient, with asterisks indicating significance levels (* *p* < 0.05, ** *p* < 0.01, *** *p* < 0.001). Each black point in the scatter plot represents one accession, with its coordinates determined by the intercept values of trait1 (x-axis) and trait2 (y-axis). (B) Enlarged scatterplots of intercept for three traits associated with yield per plant.


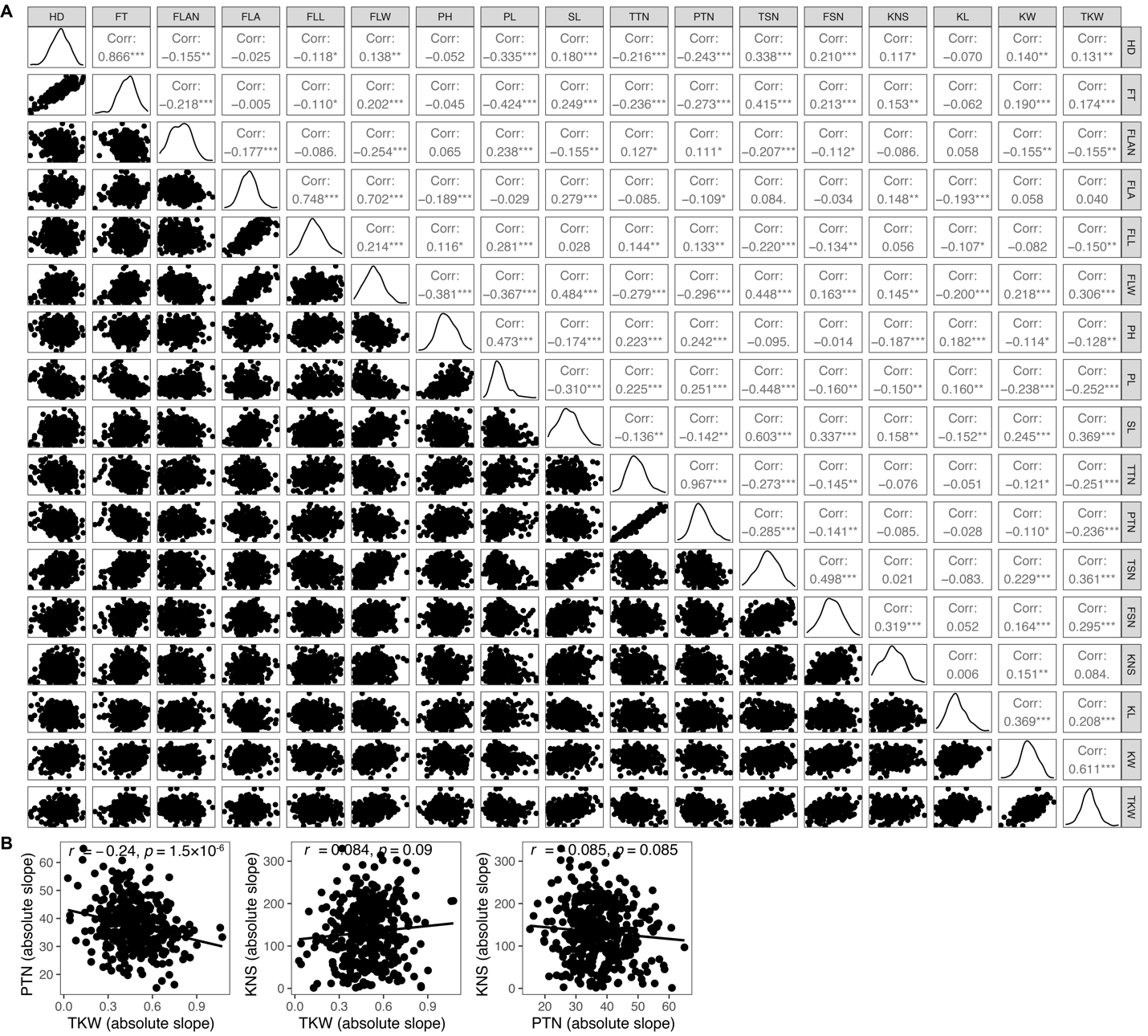


**Fig. S6. Pairwise correlation of absolute slope.** (A) Pairwise correlation of slope for 17 traits. Each cell displays the *Pearson* correlation coefficient, with asterisks indicating significance levels (* *p* < 0.05, ** *p* < 0.01, *** *p* < 0.001). Each black point in the scatter plot represents a single observation, with its coordinates determined by the slope values of trait1 (x-axis) and trait2 (y-axis). (B) Enlarged scatterplots of slope for three traits associated with yield per plant.


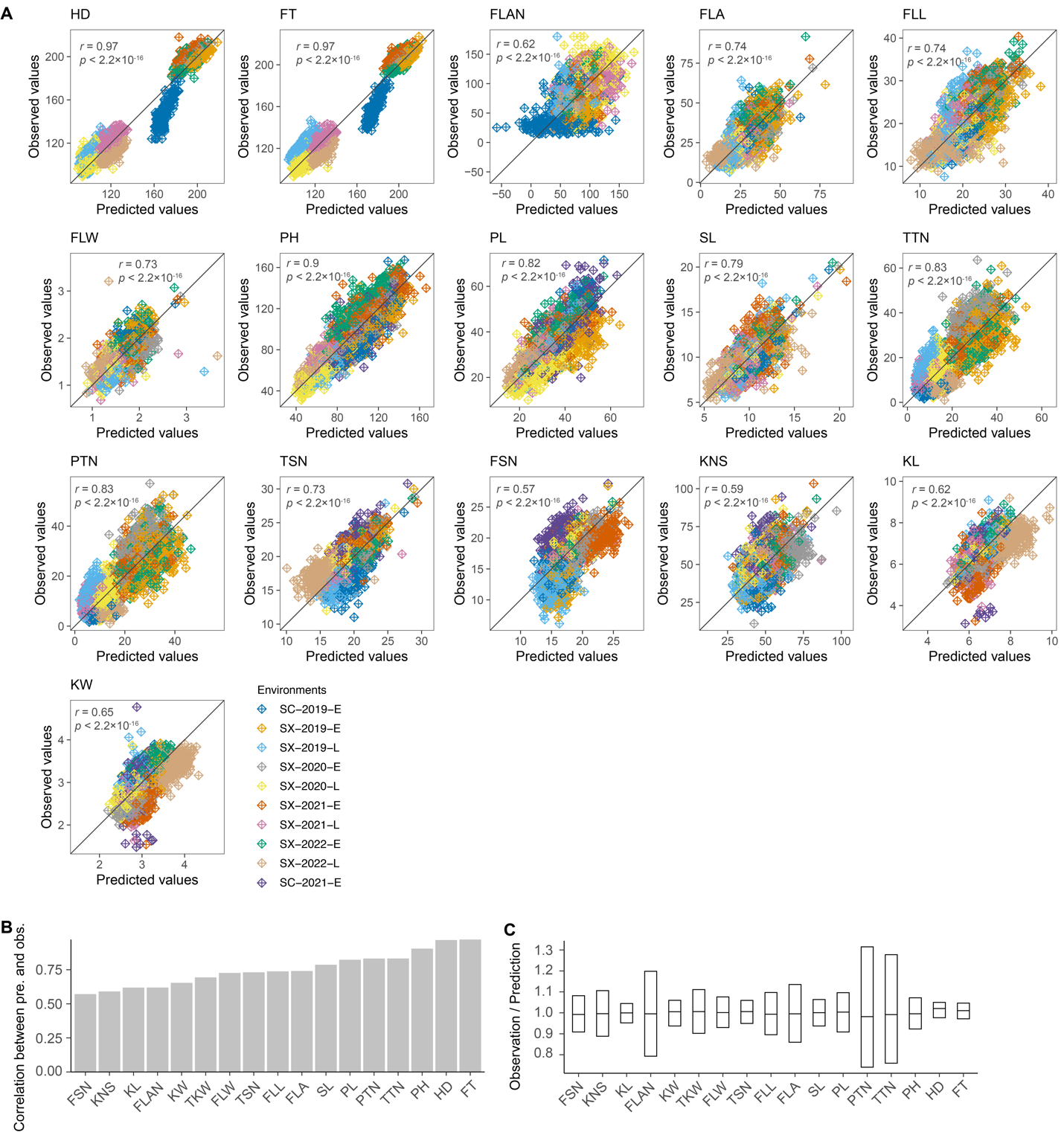


**Fig. S7. Performance predictions for tested genotypes in untested environments.** (A) Overview of predictions. Each point represents one individual and is color-coded by environment. (B, C) Correlation (B) and ratio (C) between predicted and observed values.


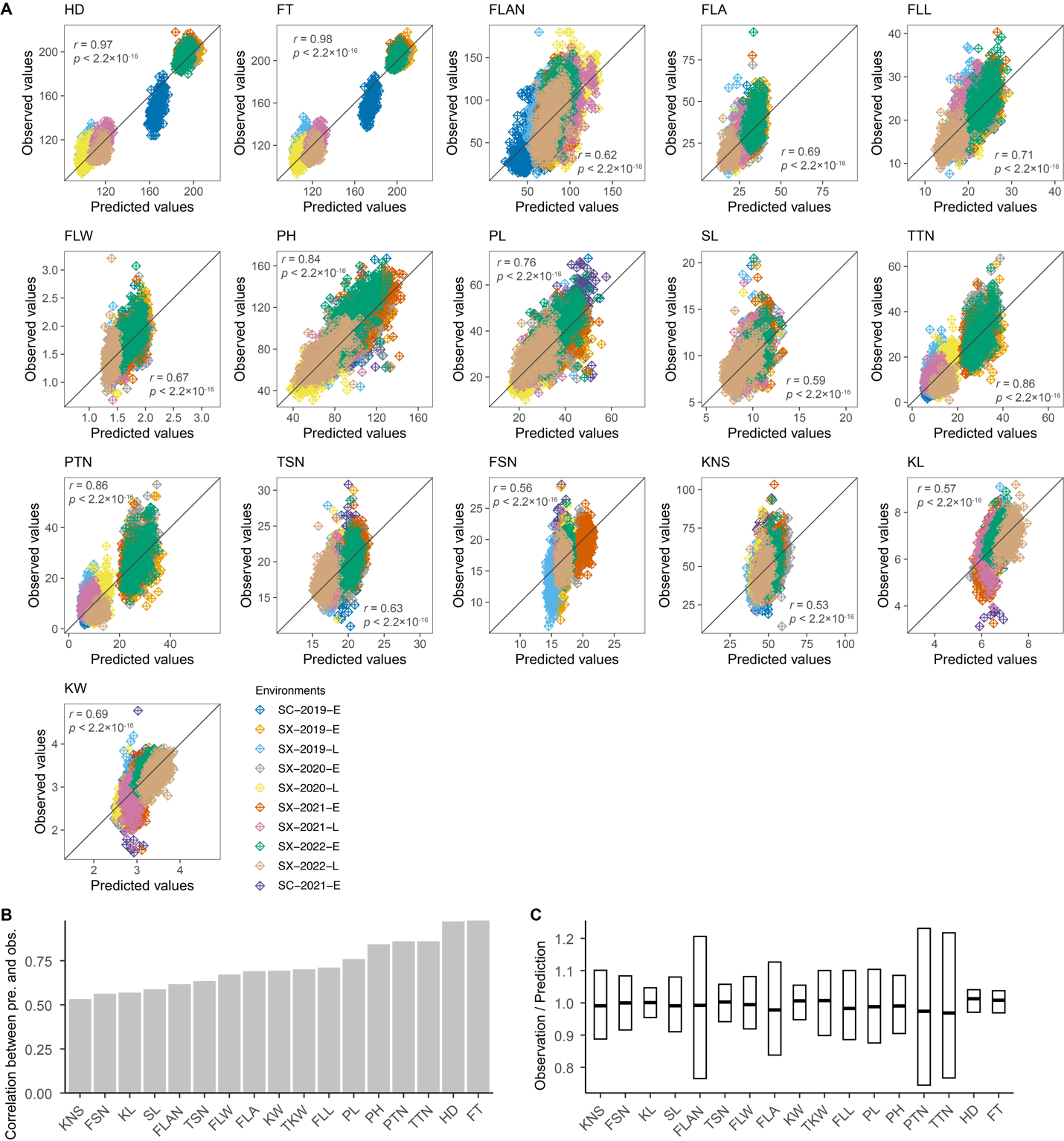


**Fig. S8. Performance predictions for untested genotypes in tested environments.** (A) Overview of predictions. Each point represents one individual and is color-coded by environment. (B, C) Correlation (B) and ratio (C) between predicted and observed values.


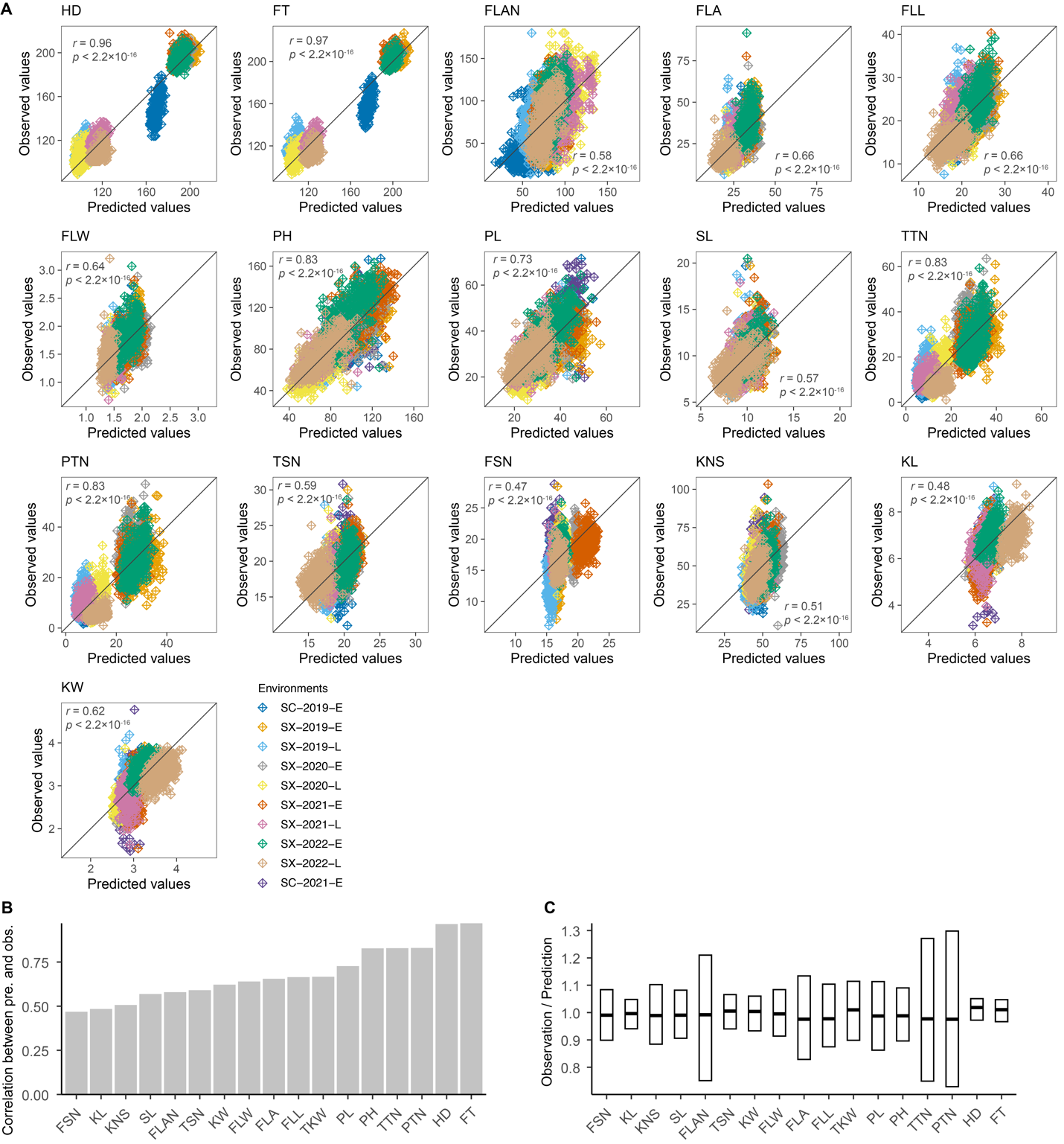


**Fig. S9. Performance predictions for untested genotypes in untested environments.** (A) Overview of predictions. Each point represents one individual and is color-coded by environment. (B, C) Correlation (B) and ratio (C) between predicted and observed values.


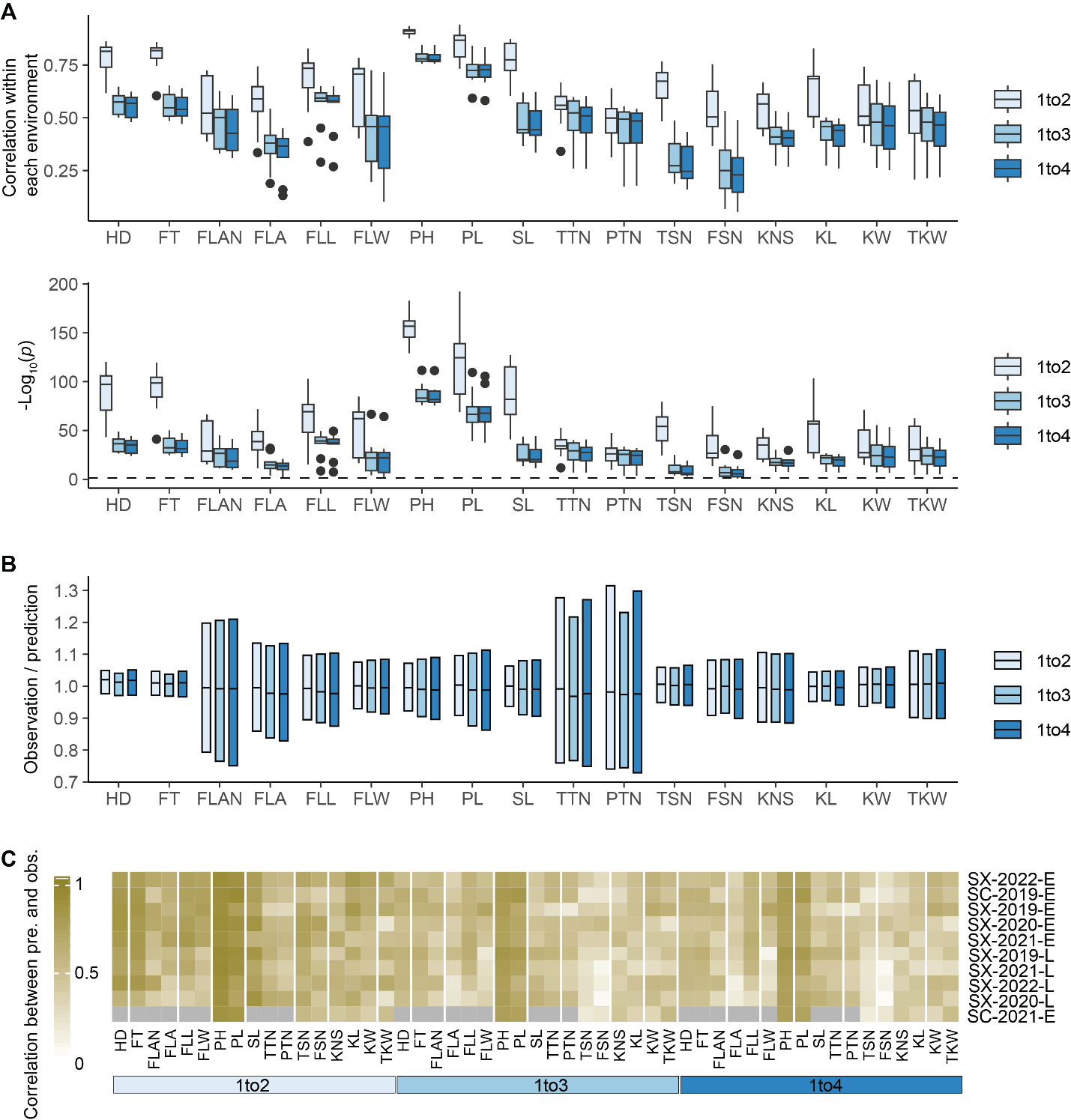


**Fig. S10. Correlation and significance between predicted and observed values within each environment.** (A) Boxplots of correlations and associated *p*-value between predicted and observed values of each trait within each environment for three prediction scenarios (1to2 represents predicting tested genotypes in untested environments, 1to3 represents predicting untested genotypes in tested environments, and 1to4 represents predicting untested genotypes in untested environments). (B) Ratios between observed and predicted values within each prediction scenario. (C) Heatmap displaying correlations between predicted and observed values in each prediction scenario. Gray indicates traits were not measured.


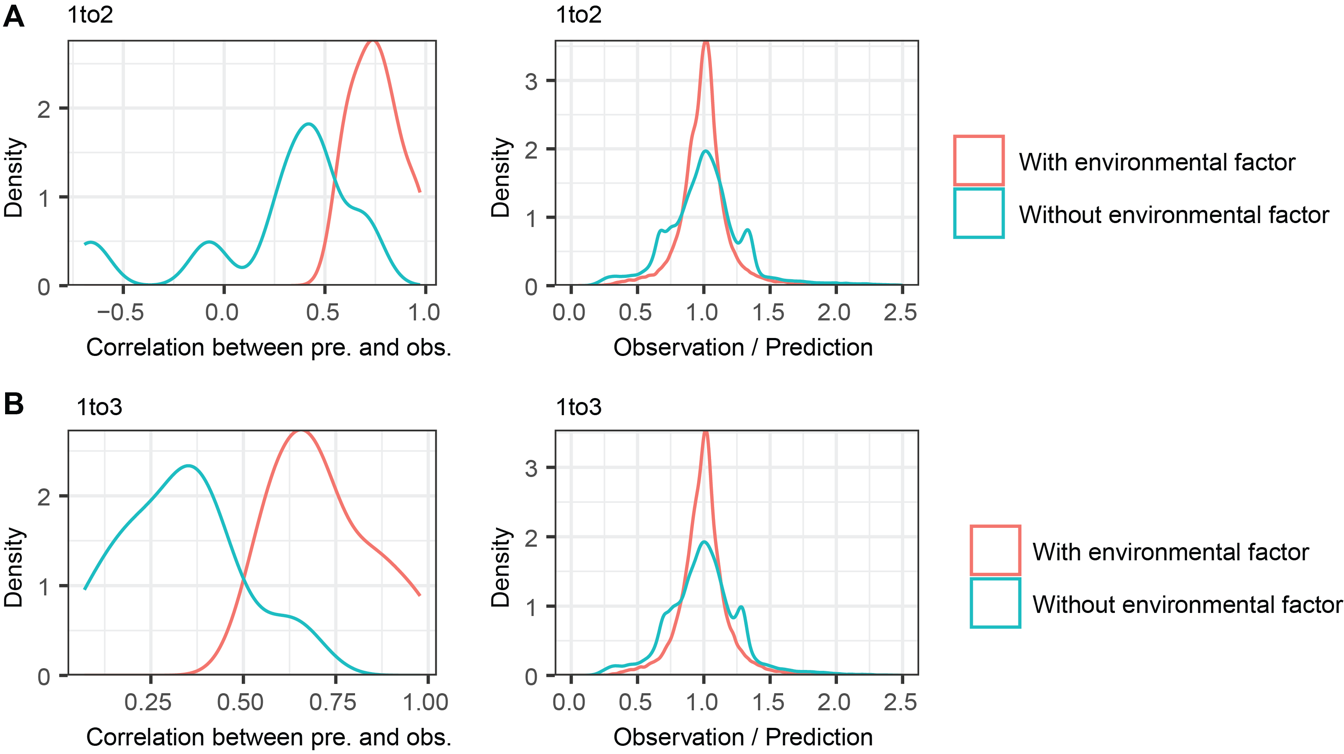


**Fig. S11. Performance predictions with and without environmental factors across 17 traits.** (A) Correlations and ratios between predicted and observed values in the 1to2 prediction scenario with and without incorporating environmental factors (1to2 represents predicting tested genotypes in untested environments). (B) Correlations and ratios between predicted and observed values in the 1to3 prediction scenario with and without incorporating environmental factors (1to3 represents predicting untested genotypes in tested environments).


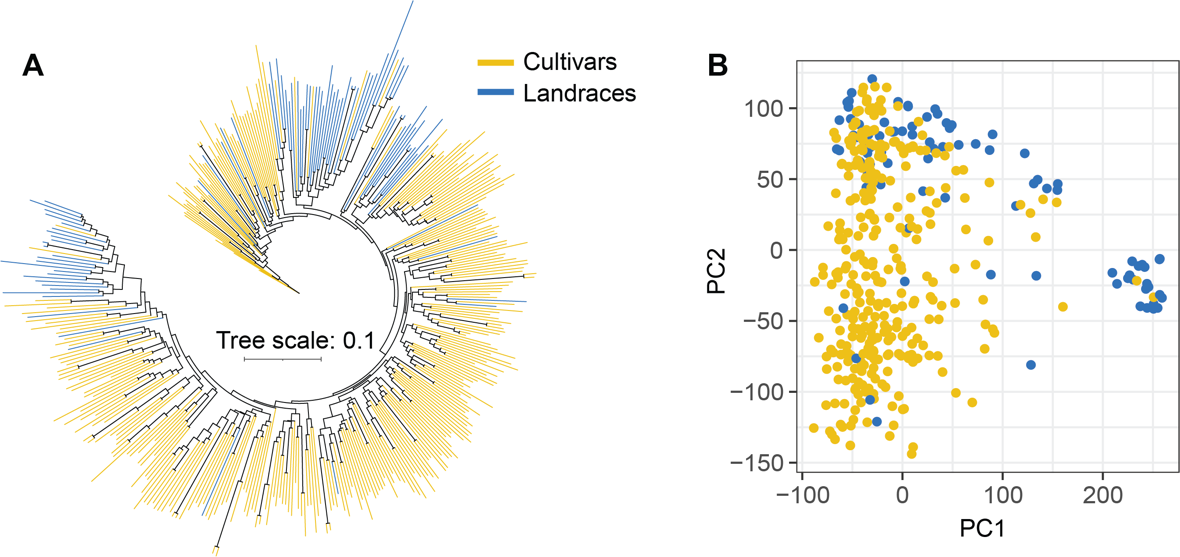


**Fig. S12. Population structure of the wheat accessions.** (A) Neighbor-joining phylogenetic tree illustrating the genetic relationships among the wheat accessions. Cultivars (yellow) and landraces (blue) are color-coded based on their classification. (B) Principal component analysis (PCA) biplot based on genome-wide SNP data, showing the genetic differentiation between cultivars and landraces along the first two principal components.


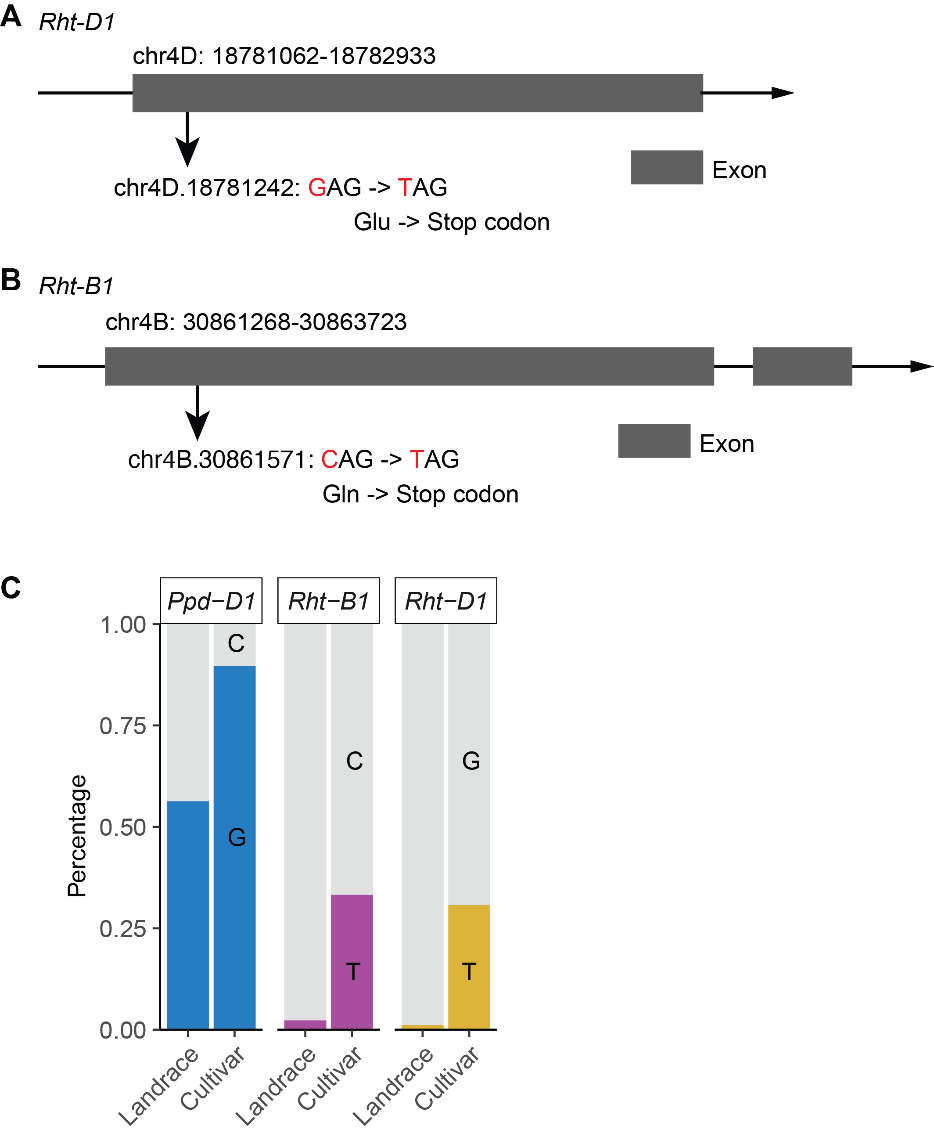


**Fig. S13. Alleles of *Rht-D1*, *Rht-B1,* and *Ppd-D1*.** (A) *Rht-D1* on chromosome 4D (18,761,062-18,782,933) contains a mutation at position 18,781,242 that resulted in a CAG (Glu) to TAG (stop codon) transition. (B) *Rht-B1* on chromosome 4B (30,861,268-30,863,723) contains a mutation at 30,861,571 that resulted in a CAG (Gln) to TAG (stop codon) transition. (C) The distribution of *Rht-B1*, *Rht-D1*, and *Ppd-D1* alleles within sub-groups.


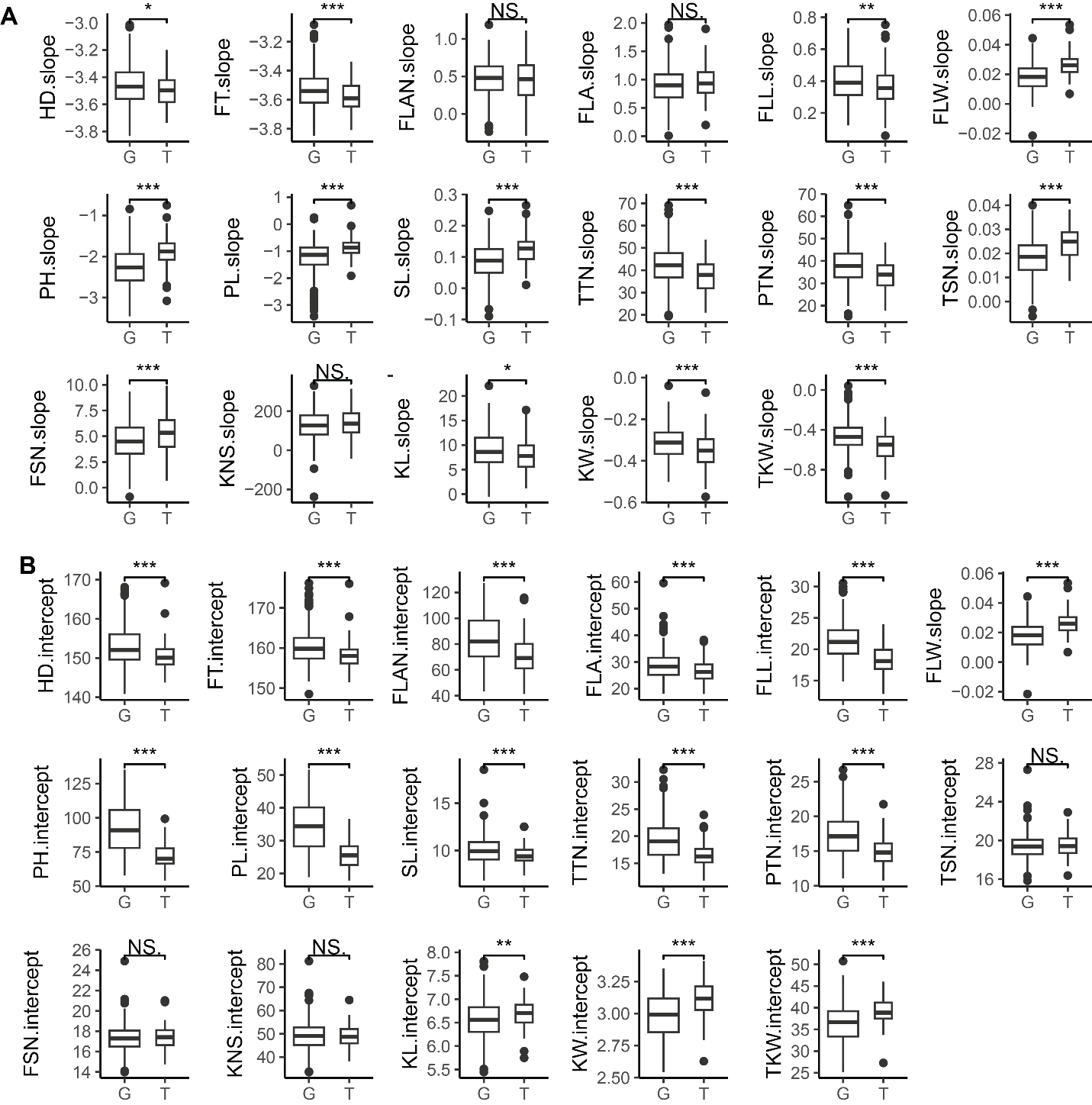


**Fig. S14. The effects of two alleles of *Rht-D1* on slope (A) and intercept (B) across 17 traits.** The T allele represents *Rht-D1b*, while the G allele represents *Rht-D1a*.


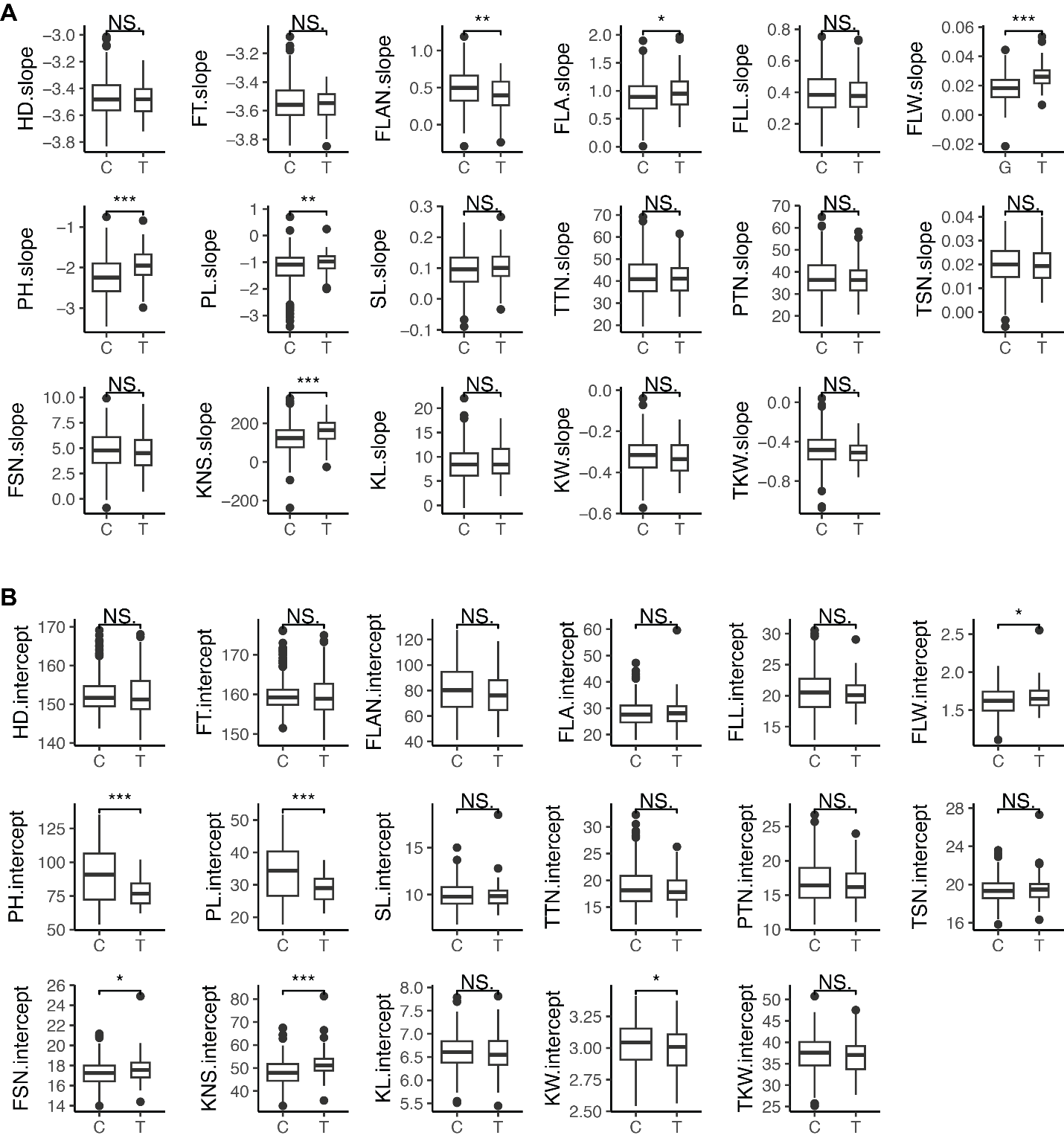


**Fig. S15. The effects of two alleles of *Rht-B1* on slope (A) and intercept (B) across 17 traits.** The T allele represents *Rht-B1b*, while the C allele represents *Rht-B1a*.


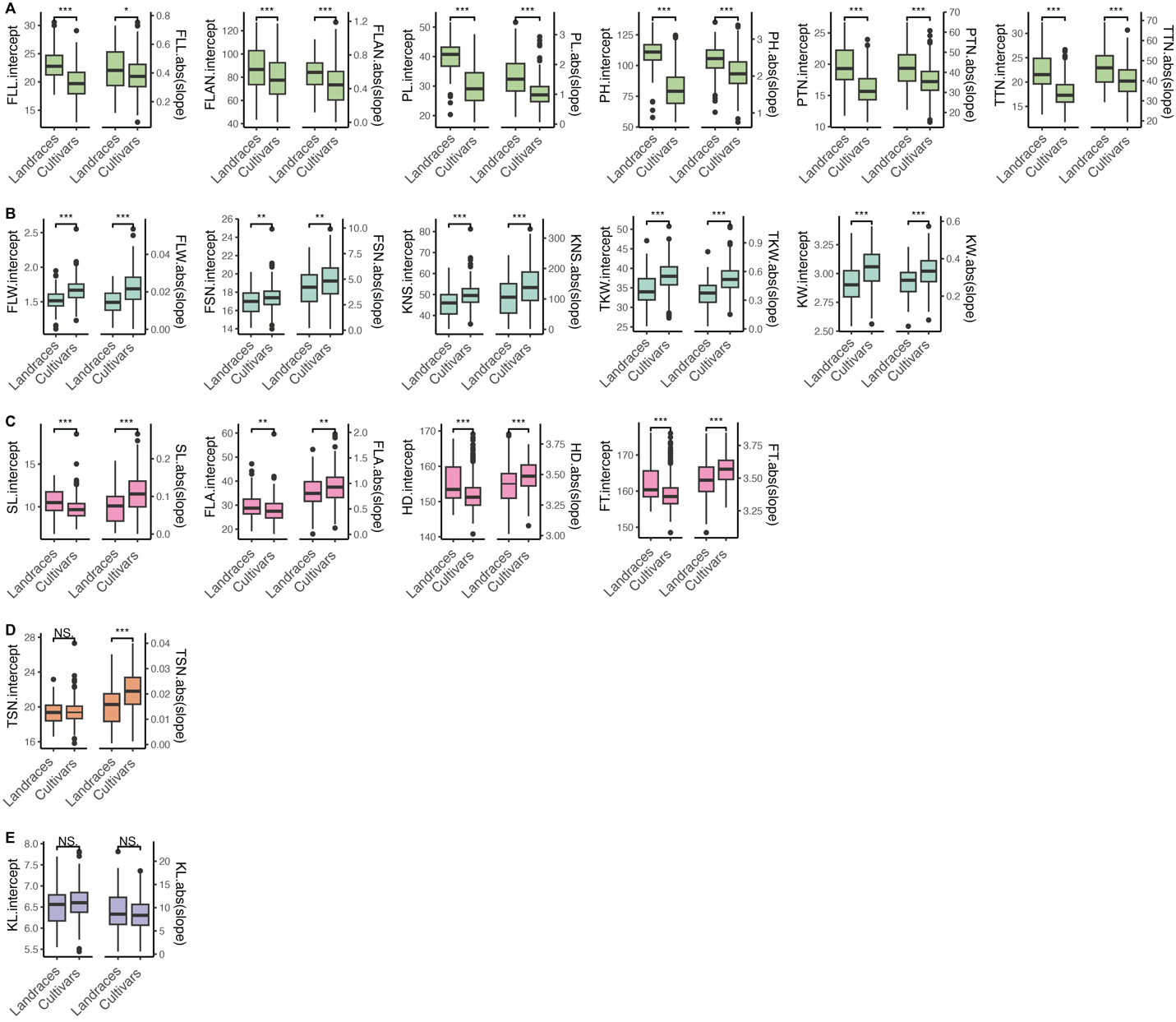


**Fig. S16. Comparison of intercept and absolute slope between landraces and cultivars across 17 traits.** Boxplots of intercept (left) and absolute slope (right) for various traits in landraces and cultivars, across five different modes (A-E). Statistical significance is indicated by asterisks (* *p* < 0.05, ** *p* < 0.01, *** *p* < 0.001), while “NS” indicates non-significant differences between landraces and cultivars.
